# Rhodium‐Mediated Stoichiometric Synthesis of Mono‐, Bi‐, and Bis‐1,2‐Azaborinines: 1‐Rhoda‐3,2‐azaboroles as Reactive Precursors

**DOI:** 10.1002/chem.202100795

**Published:** 2021-06-01

**Authors:** Merlin Heß, Ivo Krummenacher, Theresa Dellermann, Holger Braunschweig

**Affiliations:** ^1^ Institute for Inorganic Chemistry Julius-Maximilians-Universität Würzburg Am Hubland 97074 Würzburg Germany; ^2^ Institute for Sustainable Chemistry & Catalysis with Boron Julius-Maximilians-Universität Würzburg Am Hubland 97074 Würzburg Germany

**Keywords:** azaborinines, nitrogen heterocycles, cyclization, metallacycles, structure elucidation

## Abstract

A series of highly substituted 1,2‐azaborinines, including a phenylene‐bridged bis‐1,2‐azaborinine, was synthesized from the reaction of 1,2‐azaborete rhodium complexes with variously substituted alkynes. 1‐Rhoda‐3,2‐azaborole complexes, which are accessible by phosphine addition to the corresponding 1,2‐azaborete complexes, were also found to be suitable precursors for the synthesis of 1,2‐azaborinines and readily reacted with alkynyl‐substituted 1,2‐azaborinines to generate new regioisomers of bi‐1,2‐azaborinines, which feature directly connected aromatic rings. Their molecular structures, which can be viewed as boron‐nitrogen isosteres of biphenyls, show nearly perpendicular 1,2‐azaborinine rings. The new method using rhodacycles instead of 1,2‐azaborete complexes as precursors is shown to be more effective, allowing the synthesis of a wider range of 1,2‐azaborinines.

## Graphical abstract

**Boron‐nitrogen analogs of metallacyclopentadienes** in the form of 1‐rhoda‐3,2‐azaboroles (see Figure) are shown to be suitable precursors for the synthesis of 1,2‐azaborinines. Comparisons with 1,2‐azaborete rhodium complexes demonstrate their superior reactivity towards alkynes, allowing to expand the range of 1,2‐azaborinines. A series of multiply substituted 1,2‐azaborinines, including a phenylene‐bridged derivative and rare bicyclic derivatives composed of two 1,2‐azaborinine rings linked by a single bond, are reported.

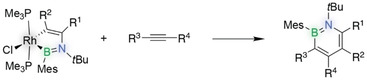

Azaborinines are heteroaromatic compounds formally derived from benzenes by replacement of two carbon atoms by one boron and one nitrogen atom.^[1]^ They are isostructural and isoelectronic to benzene and exist in three different isomeric forms depending on the relative position of the heteroatoms: 1,2‐, 1,3‐, and 1,4‐azaborinines. In 1,2‐azaborinines, the heteroatoms are joined together to form a B−N bond which, due to its polarity, imparts distinctly different properties to the structure relative to their carbon analogues.^[2]^ The replacement of arene groups with 1,2‐azaborinines in molecules thus provides a powerful means of modulating their properties.[^1^, ^2^] This concept of isosteric replacement of functional groups is widely applied in the field of medicinal research to boost the effectiveness of existing drugs or to gain new intellectual property.^[3]^ Given the prevalence of arene groups in bioactive and functional molecules, these interests have also significantly advanced the chemistry of 1,2‐azaborinines.[^1^, ^2^, ^3^, ^4^, ^5^] Over the past two decades, new and improved synthetic routes as well as selective functionalization strategies have substantially increased the diversity of available 1,2‐azaborinines.^[1]^ Beyond the typical multi‐step routes to these species, typically involving a sequence of ring‐closing metathesis and dehydrogenation, a number of conceptually different approaches enabling a more convergent construction of 1,2‐azaborinines have been developed.[^1^, ^6^]

In 2014, our group reported an efficient alternative for the synthesis of 1,2‐azaborinines based on the rhodium‐catalyzed cocyclotrimerization of iminoboranes with alkynes.^[7]^ Using iminoborane *t*BuBN*t*Bu, this protocol was limited to the synthesis of a 4,6‐diferrocenyl‐substituted derivative as small alkynes preferentially reacted to give 1,4‐azaborinine derivatives.[^7^, ^8^] The use of the unsymmetrical iminoborane MesBN*t*Bu lifted this limitation and provided corresponding 1,2‐azaborinines based on alkynes such as acetylene and phenylacetylene (Scheme 1a).^[9]^ Identification of isolable η^4^‐1,2‐azaborete complexes (**A**) as intermediates in this process allowed the synthesis of 1,2‐azaborinines in a stepwise manner, thereby significantly broadening the scope of accessible products (Scheme 1b).[^7^, ^8^, ^9^] Mechanistically, the final step of the azaborinine construction proceeds via selective alkyne insertion into the B−C bond of the azaborete ring, wherein the regioselectivity is controlled by steric effects. In analogous metal‐catalyzed cyclotrimerizations of alkynes, the corresponding cyclobutadiene complexes do not typically undergo further reaction with an alkyne to ultimately yield an arene;^[10]^ instead, metallacyclopentadienes were identified as crucial intermediates in these processes.^[11]^ Having recently isolated boron‐nitrogen derivatives of metallacyclopentadienes in the form of 1‐rhoda‐3,2‐azaborole complexes (**B**),^[12]^ we were thus intrigued to see if they could also be engaged in the synthesis of 1,2‐azaborinines (Scheme 1c). Herein, we describe our initial efforts using these rhodacycles as precursors alongside continuing efforts to map out the scope and limitations of the 1,2‐azaborinine synthesis from 1,2‐azaborete complexes.

**Scheme 1:**
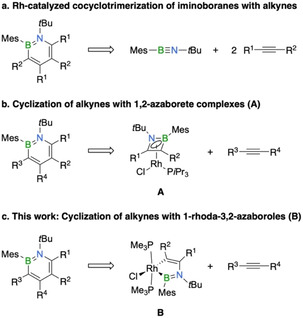
Rhodium‐based strategies for the preparation of 1,2‐azaborinines.

In order to broaden the scope of the 1,2‐azaborinine synthesis from 1,2‐azaborete complexes, we treated the rhodium η^4^‐1,2‐azaborete complex **1** with aryl‐, alkyl‐ and silyl‐substituted alkynes to give a series of 1,2‐azaborinines as shown in Scheme 2. The progress of the reactions was monitored by ^11^B NMR spectroscopy, indicating for all derivatives clean product formation. In the case of 1,2‐azaborinine **3**, multiple additions of trimethylsilylacetylene were necessary to ensure complete conversion. After purification with column chromatography, compounds **2**–**4** were obtained as pure products in moderate to good yields. Their structures were confirmed by ^1^H, ^11^B and ^13^C NMR spectroscopy: the ^11^B NMR signals between δ 37.5 and 39.5 ppm are in the typical range for 1‐*tert*‐butyl‐2‐mesityl‐1,2‐azaborinines.^[9]^ Two‐dimensional NMR spectroscopy and X‐ray crystallography unambiguously identified the azaborinines **2**–**4** as the 1,2‐isomers. While compounds **2**–**4** were obtained as colorless solids, derivative **5**, which bears six different ring substituents, was isolated as a yellow oil. Compound **5** could not be obtained in a pure form and could only be characterized by ^11^B NMR spectroscopy (δ 39.1), mass spectrometry and X‐ray crystallography. The formation of the 1,2‐azaborinines can be explained by insertion of the respective alkyne into the endocyclic B−C bond of the 1,2‐azaborete, with the sterically more demanding alkyne substituent ending up at the position β to the boron atom (i. e. the 4‐position in the six‐membered ring). The observed regiochemistry is in line with that of our previous studies on the rhodium‐mediated synthesis of 1‐*tert*‐butyl‐2‐mesityl‐4,6‐diphenyl‐1,2‐azaborinine from [2+2+2] cocyclotrimerization of iminoborane MesBN*t*Bu and phenylacetylene and the synthesis of a range of 4‐functionalized 1,2‐azaborinines from similar azaborete complexes and monosubstituted alkynes.^[9]^ X‐ray structural analysis of compounds **2**–**5** revealed typical geometries with nearly planar C_4_BN rings, B−N distances in the range of 1.453(2)–1.468(4) Å, and pronounced C−C bond length equalization (bond lengths range from 1.355(4) to 1.426(2) Å; see Figure 1).^[13]^ In agreement with previous findings,^[9]^ increasing the steric bulk next to the already congested heteroatoms by substituents in 3,6 positions results in increasing distortion of the six‐membered ring from planarity (displacements of the ring atoms from the mean C_4_BN plane: 0.03 Å (**4**) and 0.04 Å (**5**) vs. 0.005 Å (**2**) and 0.02 Å (**3**)). This is also reflected in increasing dihedral angles between the substituents on the heteroatoms: 1.9(3)° for **2**, 10.6(2)° for **3**, 11.0(4)° for **4**, and 21.8(4)° for **5**.

**Scheme 2:**
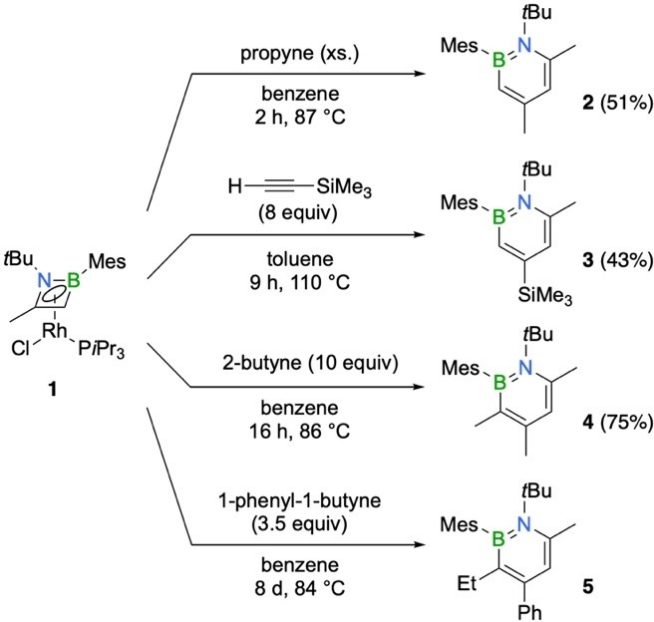
Synthesis of 1,2‐azaborinines **2**–**5** from 1,2‐azaborete complex **1** and alkynes.

**Figure 1:**
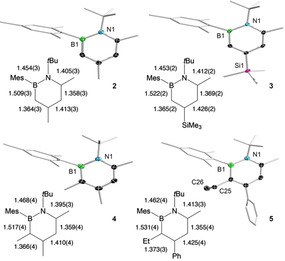
Molecular structures of 1,2‐azaborinines **2**–**5** with thermal ellipsoids for selected atoms at the 50 % probability level (hydrogen atoms not shown) and comparison of intra‐ring bond distances (Å).

We previously reported that addition of trimethylphosphine (PMe_3_) transforms the η^4^‐1,2‐azaborete complex **1** into the five‐membered 1‐rhoda‐3,2‐azaborole **6**, in which the rhodium atom is five‐coordinate with two PMe_3_ ligands and one chloride atom completing its coordination sphere (Scheme 3).^[12]^ We show now that the rhodacycle, similar to the π‐complex **1**, which was found to be an intermediate in the rhodium‐catalyzed cocyclotrimerization of alkynes with iminoboranes, can also be reacted with alkynes to generate 1,2‐azaborinines. In a one‐pot reaction, **1** was first treated with trimethylphosphine and then with trimethylsilylacetylene to afford 1,2‐azaborinine **3** in 57 % yield (Scheme 3). In contrast to the direct reaction of **1** with the alkyne, formation of the 1,2‐azaborinine proceeds at room temperature, showing that addition of PMe_3_ to **1** and thus the formation of complex **6** facilitates the cyclization. Encouraged by the success of the reaction and the observed high propensity of rhodacycles to mediate alkyne cyclization, we became interested in further exploring the potential of these complexes for 1,2‐azaborinine synthesis.

**Scheme 3:**
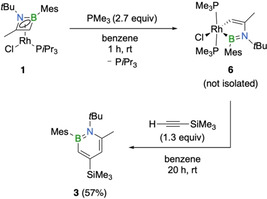
Synthesis of 1,2‐azaborinine **3** via the 1‐rhoda‐3,2‐azaborole intermediate **6**.

As shown in Scheme 4, we successfully employed these five‐membered rhodacycles as precursors for the synthesis of a new family of 1,2‐azaborinine dimers consisting of two 1,2‐azaborinine units connected by a single C−C bond via their respective 4 and 5’ positions. The dimers were obtained by the reaction of the rhodacycles **6** and **9**,^[12]^ respectively, with the alkynyl‐substituted 1,2‐azaborinine **7** (Scheme 4).^[12]^ The end of the reaction was indicated by complete conversion of **7**, which was readily verified by ^1^H NMR spectroscopy. Alongside the 1,2‐azaborinine products, we observed the formation of the rhodium(I) chloride dimer [RhCl(PMe_3_)_2_]_2_ as indicated by the ^31^P NMR signal at δ (^31^P)=3.7 ppm with a characteristic rhodium‐phosphorus coupling constant of ^1^ *J*_RhP_=191 Hz.^[14]^ Following workup, the 4,5’‐bi‐1,2‐azaborinine products **8** and **10** were isolated as colorless solids in yields of 60 % and 38 %, respectively. The compounds are characterized by a single ^11^B NMR signal at 38.4 ppm (**8** and **10**) and characteristic ^1^H NMR signals for the azaborinine protons (**8**: δ 6.28, 6.68 and 7.46 ppm; **10**: δ 6.67 and 7.40 ppm). In each case, only one isomer is formed, with the regiochemistry controlled by steric effects. The crystal structures of **8** and **10**,^[13]^ as shown in Figure 2, indicate that the 1,2‐azaborinine units are considerably twisted relative to each other, with the rings in **10** oriented nearly perfectly perpendicular to each other (torsion angle of 89° in **10** vs. 74° in **8**). While the heterocyclic rings are only moderately distorted in **10**, as indicated by relatively small exocyclic C−N−B−C dihedral angles of about 10°, the more extensively substituted 1,2‐azaborinine moiety in **8** is considerably strained, with a corresponding angle of 22.1(2)°. The ring bond distances, which are displayed in Figure 2, display no unusual features. Oligomers of 1,2‐azaborinines have been reported by the group of Liu and Jäkle.^[15]^ Thereby, through Suzuki‐Miyaura cross‐coupling, monomeric 1,2‐azaborinine units were joined together via their 3 and 6 positions, affording dimers, trimers and even a polymer. Due to their different connectivity, **8** and **10** can be regarded as new regioisomers of bi‐1,2‐azaborinines.

**Scheme 4:**
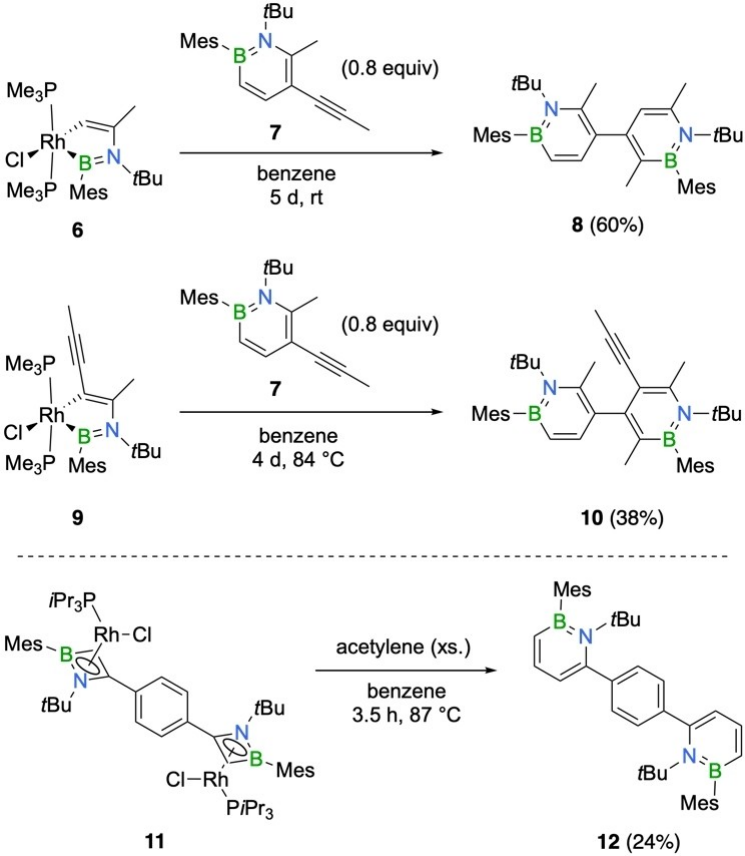
Synthesis of unbridged and *p*‐phenylene‐bridged 1,2‐azaborinines.

**Figure 2:**
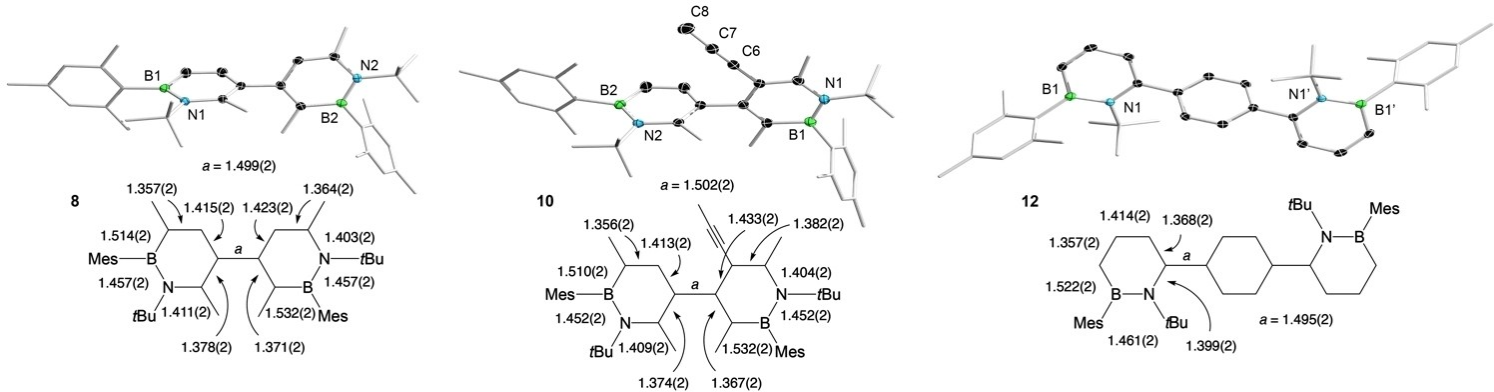
Molecular structures of the 1,2‐azaborinines **8**, **10** and **12** with thermal ellipsoids for selected atoms at the 50 % probability level (hydrogen atoms are not shown). Symmetry‐equivalent atoms for **12** are labeled with a prime. Intra‐ring bond distances (Å) are indicated below the structures.

Notably, the rhodium π‐complex **1** did not react with the alkynyl‐substituted 1,2‐azaborinine **7** under similar conditions, indicating that the rhodacycles are less sensitive to steric hindrance than the azaborete complexes. Given their high reactivity toward alkyne cyclization, these rhodacycles may thus prove to be superior precursors for the synthesis of 1,2‐azaborinines, especially highly substituted and sterically hindered derivatives. A more comprehensive and systematic study of their reactivity will follow.

Using the established protocol starting from 1,2‐azaborete complexes, we also synthesized a bridged 1,2‐azaborinine derivative. Treatment of the previously reported dinuclear 1,2‐azaborete complex **11** ^[12]^ with an excess of acetylene in refluxing benzene afforded the p‐phenylene‐bridged 1,2‐azaborinine dimer **12** in 24 % yield after purification by silica gel chromatography (Scheme 4). Its structure was confirmed by NMR spectroscopy, single‐crystal X‐ray crystallography and high‐resolution mass spectrometry. Its solid‐state structure shows that the adjacent aromatic rings are twisted out of plane with a dihedral C−C−C−C angle of 55.5(2)°. The two 1,2‐azaborinine groups are arranged in a *trans*‐*anti* fashion. With a torsion angle between the B,N‐substituents of 27.3(2)°, the 1,2‐azaborinine units are highly strained and distorted, as is to be expected from the presence of two large substituents next to nitrogen. In solution, bis‐1,2‐azaborinine **12** exists as *trans* and *cis* atropisomers, as indicated by two inequivalent environments for the *tert*‐butyl groups (δ (^1^H)=1.35 and 1.33 ppm in a nearly 1 : 1 ratio) and two sets of three signals for the 1,2‐azaborinine ring protons (δ (^1^H)=7.40, 6.79, 6.18 and 6.13 ppm; two signals of each set overlap). However, the broadness of the ^11^B NMR spectroscopic signal of **12** (δ 40.8 ppm) does not allow us to distinguish between the two isomers. Having *tert*‐butyl substituents at *ortho* positions of the two azaborinine units, compound **12** is expected to display atropisomerism due to the hindered rotation about the biphenyl axis.^[16]^ We have identified similar atropisomers for a closely related derivative containing 1,4‐ instead of 1,2‐azaborinine units.^[8b]^ By contrast, the linear 1,2‐azaborinine trimer reported by the groups of Liu and Jäkle preferentially adopts a *syn* disposition due to favorable N−H⋅⋅⋅π interactions.^[15]^ The presence of a similar interaction in a diphenylacetylene analogue also favored the *syn* arrangement of the 1,2‐azaborinine groups.^[17]^

In summary, we have prepared a series of highly substituted 1,2‐azaborinine derivatives, including a diaza/dibora analogue of *p*‐terphenyl, by reaction of η^4^‐1,2‐azaborete rhodium complexes with a range of mono‐ and disubstituted alkynes. Presumably as a result of alleviating steric strain from interactions with the bulky substituents on the heteroatoms, the 3‐functionalized 1,2‐azaborinines show the largest departures from planarity. Interestingly, 1‐rhoda‐3,2‐azaboroles, which are derived from rhodium 1,2‐azaborete complexes by addition of phosphine, were found to be more effective in mediating the cyclization to 1,2‐azaborinines than the 1,2‐azaborete complexes. Their higher reactivity was revealed by the synthesis of two 1,2‐azaborinine dimers with a new 4,5’ connectivity that proved to be inaccessible by employing corresponding 1,2‐azaborete complexes as precursors. Broadening the scope of the 1,2‐azaborinine synthesis from these metallacycles will be the subject of future studies.

## Conflict of interest

The authors declare no conflict of interest.

## Supporting information

As a service to our authors and readers, this journal provides supporting information supplied by the authors. Such materials are peer reviewed and may be re‐organized for online delivery, but are not copy‐edited or typeset. Technical support issues arising from supporting information (other than missing files) should be addressed to the authors.

Supplementary
