# Supplementary material for: Rhodium‐Mediated Stoichiometric Synthesis of Mono‐, Bi‐, and Bis‐1,2‐Azaborinines: 1‐Rhoda‐3,2‐azaboroles as Reactive Precursors
Source: Chemistry. 2021 Jun 1;27(37):9503–7. doi: 10.1002/chem.202100795 (PMC8362125; doi:10.1002/chem.202100795)
Supplement: Supplementary file 1 — Supplementary [file CHEM-27-9503-s001.pdf]

Chemistry–A European Journal

Supporting Information

Rhodium-Mediated Stoichiometric Synthesis of Mono-, Bi-, and Bis-1,2-Azaborinines: 1-Rhoda-3,2-azaboroles as Reactive Precursors

Merlin Heß, Ivo Krummenacher, Theresa Dellermann, and Holger Braunschweig*

Table of Contents

General Considerations 1

Synthetic Procedures 2

NMR Spectra 9

Crystal Structure Determination 20

References 23

General Considerations

Except for column chromatography, all manipulations were performed under an atmosphere of dry argon using glovebox or standard Schlenk line techniques. Deuterated solvents were dried over 4 Å molecular sieves and degassed by three freeze-pump-thaw cycles. All other solvents were dried by distillation from appropriate drying agents under an argon atmosphere and stored under argon over activated 4 Å molecular sieves. All NMR spectra were obtained from a Bruker Avance I 400 NMR spectrometer (^{11}B: 128.5 MHz) or from a Bruker Avance I 500 NMR spectrometer (^{1}H: 500.1 MHz, ^{13}C{^{1}H}: 125.8 MHz, ^{11}B: 160.5 MHz, ^{29}Si: 99.4 MHz). Chemical shifts (δ) are provided in ppm and internally referenced to the carbon nuclei (^{13}C{^{1}H}) or residual protons (^{1}H) of the solvent. ^{11}B NMR and ^{29}Si NMR spectra were referenced against external $BF_3 \cdot Et_2O$ and $SiMe_4$, respectively. High-resolution mass spectrometry data were acquired on a Thermo Scientific Exactive Plus Spectrometer in ASAP mode. Elemental analysis was conducted on an Elementar vario MICRO cube elemental analyser.

[RhCl(η^4-1-*tert*-butyl-2-mesityl-4-methyl-1,2-azaborete)(P*i*Pr$_3$)] (**1**),[1] 1-rhoda-2-mesityl-3-*tert*-butyl-4-methyl-3,2-azaborole **6**,[1] 1-*tert*-butyl-2-mesityl-6-methyl-5-(prop-1-ynyl)-1,2-azaborinine (**7**),[1] 1-rhoda-2-mesityl-3-*tert*-butyl-4-methyl-5-(prop-1-ynyl)-3,2-azaborole **9**,[1] the rhodium bis(azaborete) complex **11**,[1] and trimethylphosphine[2] were synthesized according to modified literature procedures. All other chemicals were purchased from either abcr, Acros, Sigma-Aldrich or TCI Chemical Co. and used without further purification. The new azaborinines 1-*tert*-butyl-2-mesityl-4,6-dimethyl-1,2-azaborinine (**2**), 1-*tert*-butyl-2-mesityl-6-methyl-4-trimethylsilyl-1,2-azaborinine (**3**), 1-*tert*-butyl-2-mesityl-3,4,6-trimethyl-1,2-azaborinine (**4**), 1-*tert*-butyl-3-ethyl-2-mesityl-6-methyl-4-phenyl-1,2-azaborinine (**5**), and the *p*-phenylene-bridged bis-1,2-azaborinine **12** were synthesized according to a standardized procedure adapted from a previously published route.[3]

Synthetic Procedures

Synthesis of 1-*tert*-butyl-2-mesityl-4,6-dimethyl-1,2-azaborinine (**2**)

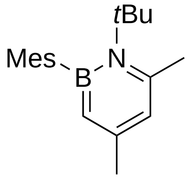

[RhCl(η^4-1-*tert*-butyl-2-mesityl-4-methyl-1,2-azaborete)(P*i*Pr_3)] (**1**)[1] (225 mg, 417 µmol) was dissolved in benzene (8 mL) and the argon atmosphere was replaced by propyne. After stirring the reaction mixture for 2 h at 87 °C, all volatiles were removed *in vacuo*. The residue was purified by column chromatography on silica gel with a mixture of hexane and diethyl ether (50:1) as eluent. Evaporation of the product-containing fractions afforded pure **2** as a colorless solid (60.0 mg, 213 µmol, 51%). Crystals of **2** suitable for X-ray diffraction were obtained by evaporation of a saturated pentane solution at −30 °C.

^{1}H NMR (500.1 MHz, C_6D_6, 298 K): δ = 6.89-6.88 (m, 2H, Mes-C*H*), 6.348-6.346 (m, 1H, Aza-C*H*), 6.00 (d, $^4J_{HH}$ = 2.7 Hz, 1H, Aza-C*H*), 2.34 (s, 3H, Aza-CH_3), 2.291 (s, 6H, Mes-CH_3), 2.285 (s, 3H, Mes-CH_3), 2.065-2.063 (m, 3H, Aza-CH_3), 1.34 (s, 9H, *t*Bu-CH_3) ppm.

^{13}C{^{1}H} NMR (125.8 MHz, C_6D_6, 298 K): δ = 151.5 (s, Aza-C_q), 148.4 (detected by HMBC, Mes-C_q), 145.7 (s, Aza-C_q), 136.8 (s, Mes-C_q), 135.1 (s, Mes-C_q), 131.4 (br s, Aza-*C*H), 127.7 (s, Mes-*C*H), 119.5 (s, Aza-*C*H), 60.7 (s, *t*Bu-C_q), 34.5 (s, *t*Bu-CH_3), 26.7 (s, Aza-CH_3), 24.1 (s, Mes-CH_3), 23.5 (s, Aza-CH_3), 21.4 (s, Mes-CH_3) ppm.

^{11}B NMR (160.5 MHz, C_6D_6, 298 K): δ = 38.5 (br s) ppm.

Elemental analysis: % *calcd*: C 81.14, H 10.04, N 4.98; *found*: C 80.51, H 9.95, N 4.71.

HRMS (ASAP, $C_{19}H_{28}BN$ + H): *calcd*: m/z = 282.2388; *found*: m/z = 282.2383.

Synthesis of 1-*tert*-butyl-2-mesityl-6-methyl-4-trimethylsilyl-1,2-azaborinine (**3**)

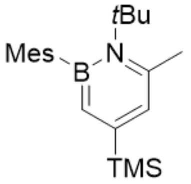

Method A: [RhCl(η^4-1-*tert*-butyl-2-mesityl-4-methyl-1,2-azaborete)(P*i*Pr$_3$)] (**1**)[1] (1.27 g, 2.35 mmol) and trimethylsilylacetylene (461 mg, 0.65 mL, 4.69 mmol) were dissolved in toluene (15 mL) and the reaction mixture was stirred for 3 h at 110 °C. Trimethylsilylacetylene (461 mg, 0.65 mL, 4.69 mmol) was added again and the reaction mixture was stirred for 2 h at 110 °C. This process was repeated two more times and then all volatiles were removed in vacuo. The residue was purified three times by column chromatography on silica gel with a mixture of pentane and diethyl ether (100:1) as eluent. Evaporation of the product-containing fractions afforded pure **3** as a colorless solid (343 mg, 1.01 mmol, 43%). Crystals of **3** suitable for X-ray diffraction were obtained by evaporation of a saturated pentane solution at −30 °C.

Method B: [RhCl(η^4-1-*tert*-butyl-2-mesityl-4-methyl-1,2-azaborete)(P*i*Pr$_3$)] (**1**)[1] (1.29 g, 2.39 mmol) was dissolved in benzene (50 mL) and treated with a stock solution of trimethylphosphine in benzene (7.18 mL, 6.37 mmol, 0.887 M). After stirring the reaction mixture for 1 h at room temperature, all volatiles were removed *in vacuo*. The residue was suspended twice in hexane (2 x 20 mL) and dried under reduced pressure to yield 1-rhoda-2-mesityl-3-*tert*-butyl-4-methyl-3,2-azaborole **6**[1] as a yellow solid. The solid was dissolved in benzene (20 mL) and trimethylsilylacetylene (235 mg, 0.34 mL, 2.39 mmol) was added. After stirring the reaction mixture for 17 h at room temperature, trimethylsilylacetylene (69 mg, 0.1 mL, 703 µmol) was added again, the reaction mixture was stirred for a further 3 h at ambient temperature and all volatiles were removed *in vacuo*. The residue was purified by column chromatography on silica gel with hexane as eluent. Evaporation of the product-containing fractions afforded pure **3** as a colorless solid (463 mg, 1.36 mmol, 57%).

^{1}H NMR (500.1 MHz, C_6D_6, 298 K): δ = 7.024-7.021 (m, 1H, Aza-C*H*), 6.884-6.881 (m, 2H, Mes-C*H*), 6.51-6.50 (m, 1H, Aza-C*H*), 2.41 (s, 3H, Aza-CH_3), 2.283 (s, 6H, Mes-CH_3), 2.278 (s, 3H, Mes-CH_3), 1.34 (s, 9H, *t*Bu-CH_3), 0.21 (s, 9H, TMS-CH_3) ppm.

^{13}C{^{1}H} NMR (125.8 MHz, C_6D_6, 298 K): δ = 156.1 (s, Aza-C_q), 148.3 (br s, Mes-C_q), 144.2 (s, Aza-C_q), 141.4 (br s, Aza-*C*H), 136.9 (s, Mes-C_q), 135.3 (s, Mes-C_q), 127.7 (s, Mes-*C*H), 120.7 (s, Aza-*C*H), 61.1 (s, *t*Bu-C_q), 34.4 (s, *t*Bu-*C*H$_3$), 27.0 (s, Aza-*C*H$_3$), 24.2 (s, Mes-*C*H$_3$), 21.3 (s, Mes-*C*H$_3$), −1.35 (s, TMS-*C*H$_3$) ppm.

^{11}B NMR (160.5 MHz, C_6D_6, 298 K): δ = 37.5 (br s) ppm.

^{29}Si NMR (99.4 MHz, C_6D_6, 298 K): δ = −5.7 (s) ppm.

Elemental analysis: % *calcd*: C 74.32, H 10.10, N 4.13; *found*: C 73.93, H 10.12, N 4.22.

HRMS (ASAP, $C_{21}H_{34}BNSi$ + H): *calcd*: m/z = 340.2626; *found*: m/z = 340.2624.

Synthesis of 1-*tert*-butyl-2-mesityl-3,4,6-trimethyl-1,2-azaborinine (**4**)

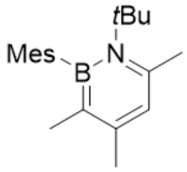

[RhCl(η^4-1-*tert*-butyl-2-mesityl-4-methyl-1,2-azaborete)(P*i*Pr_3)] (**1**)[1] (630 mg, 1.17 mmol) and 2-butyne (633 mg, 11.7 mmol) were dissolved in benzene (10 mL) and the reaction mixture was stirred for 16 h at 86 °C. All volatiles were removed in vacuo and the residue was purified by column chromatography on silica gel with a mixture of pentane and diethyl ether (50:1) as eluent. Evaporation of the product-containing fractions afforded pure **4** as a colorless solid (260 mg, 881 µmol, 75%). Crystals of **4** suitable for X-ray diffraction were obtained by evaporation of a saturated pentane solution at −30 °C.

^{1}H NMR (500.1 MHz, C_6D_6, 298 K): δ= 6.88 (s, 2H, Mes-C*H*), 6.08 (s, 1H, Aza-C*H*), 2.36 (s, 3H, Aza-CH_3), 2.29 (s, 3H, Mes-CH_3), 2.20 (s, 6H, Mes-CH_3), 2.05 (s, 3H, Aza-CH_3), 1.78 (s, 3H, Aza-CH_3), 1.34 (s, 9H, *t*Bu-CH_3) ppm.

^{13}C{^{1}H} NMR (125.8 MHz, C_6D_6, 298 K): δ = 147.7 (s, Aza-C_q), 147.2 (detected by HMBC, Mes-C_q), 142.2 (s, Aza-C_q), 137.3 (s, Mes-C_q), 135.3 (s, Mes-C_q), 134.9 (detected by HMBC, Aza-C_q), 127.7 (s, Mes-*C*H), 120.8 (s, Aza-*C*H), 60.8 (s, *t*Bu-C_q), 34.7 (s, *t*Bu-*C*H_3), 26.8 (s, Aza-*C*H_3), 23.4 (s, Mes-*C*H_3), 21.4 (s, Mes-*C*H_3), 20.0 (s, Aza-*C*H_3), 16.8 (s, Aza-*C*H_3) ppm.

^{11}B NMR (160.5 MHz, C_6D_6, 298 K): δ = 38.7 (br s) ppm.

Elemental analysis: % *calcd*: C 81.35, H 10.24, N 4.74; *found*: C 80.65, H 10.50, N 4.45.

HRMS (ASAP, $C_{20}H_{30}BN$ + H): *calcd*: m/z = 296.2544; *found*: m/z = 296.2538.

Synthesis of 1-*tert*-butyl-3-ethyl-2-mesityl-6-methyl-4-phenyl-1,2-azaborinine (**5**)

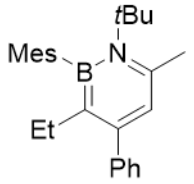

[RhCl(η^4-1-*tert*-butyl-2-mesityl-4-methyl-1,2-azaborete)(P*i*Pr$_3$)] (**1**)[1] (392 mg, 726 µmol) and 1-phenyl-1-butyne (142 mg, 0.16 mL, 1.09 mmol) were dissolved in benzene (8 mL) and the reaction mixture was stirred for 6 d at 84 °C. 1-Phenyl-1-butyne (189 mg, 0.21 mL, 1.45 mmol) was added again and the reaction mixture was stirred for 2 d at 84 °C. All volatiles were removed in vacuo and the residue was purified by column chromatography on silica gel with a mixture of hexane and dichloromethane (20:1) as eluent. Evaporation of the product-containing fractions afforded a yellow oil, which was found to be analytically impure. Crystals of **5** suitable for X-ray diffraction were obtained by evaporation of a saturated pentane solution at −30 °C.

^{11}B NMR (128.4 MHz, C_6D_6, 298 K): δ = 39.1 (br s) ppm.

HRMS (ASAP, $C_{26}H_{34}BN$ + H): *calcd*: m/z = 372.2857, *found*: m/z = 372.2850.

Synthesis of 4,5'-bi-1,2-azaborinine **8**

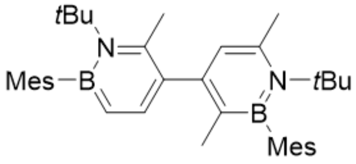

1-Rhoda-2-mesityl-3-*tert*-butyl-4-methyl-3,2-azaborole complex **6**[1] (80.0 mg, 150 µmol) and 1-*tert*-butyl-2-mesityl-6-methyl-5-(prop-1-ynyl)-1,2-azaborinine (**7**)[1] (35.3 mg, 116 µmol) were dissolved in benzene (8 mL) and the reaction mixture was stirred for 5 d at room temperature. After removing all volatiles *in vacuo*, the residue was purified by column chromatography on silica gel with a mixture of hexane and diethyl ether (50:1) as eluent. Evaporation of the solvent from the first fractions yielded pure bi-1,2-azaborinine **8** as a colorless solid (38.0 mg, 69.5 µmol, 60%). Crystals of **8** suitable for X-ray diffraction were obtained by evaporation of a saturated hexane/diethyl ether (50:1) solution.

^{1}H NMR (500.1 MHz, C_6D_6, 298 K): δ = 7.46 (d, $^3J_{HH}$ = 10.8 Hz, 1H, Aza-C*H*), 6.92 (s, 1H, Mes-C*H*), 6.88 (s, 2H, Mes-C*H*), 6.87 (s, 1H, Mes-C*H*), 6.68 (d, $^3J_{HH}$ = 10.8 Hz, 1H, Aza-C*H*), 6.28 (s, 1H, Aza-C*H*), 2.46 (s, 3H, Aza-CH_3), 2.36 (s, 3H, Aza-CH_3), 2.35 (s, 3H, Mes-CH_3),

2.287-2.285 (m, 6H, Mes-CH_3), 2.27 (s, 3H, Mes-CH_3), 2.251 (s, 3H, Mes-CH_3), 2.248 (s, 3H, Mes-CH_3), 1.72 (s, 3H, Aza-CH_3), 1.42 (s, 9H, *t*Bu-CH_3), 1.37 (s, 9H, *t*Bu-CH_3) ppm.

^{13}C{^{1}H} NMR (125.8 MHz, C_6D_6, 298 K): δ = 154.2 (s, Aza-C_q), 147.9 (detected by HMBC, Mes-C_q), 147.1 (detected by HMBC, Mes-C_q), 143.7 (s, Aza-*C*H), 143.1 (s, Aza-C_q), 142.2 (s, Aza-C_q), 137.1 (s, Mes-C_q), 137.0 (s, Mes-C_q), 136.82 (s, Mes-C_q), 136.76 (s, Mes-C_q), 135.7 (detected by HMBC, Aza-C_q), 135.4 (s, Mes-C_q), 135.3 (s, Mes-C_q), 132.2 (br s, Aza-*C*H), 129.5 (s, Aza-C_q), 127.9 (detected by HSQC, Mes-*C*H), 127.8 (detected by HSQC, Mes-*C*H), 127.7 (s, Mes-*C*H), 119.8 (s, Aza-*C*H), 61.2 (s, *t*Bu-C_q), 61.1 (s, *t*Bu-C_q), 34.8 (s, *t*Bu-CH_3), 34.7 (s, *t*Bu-CH_3), 26.9 (s, Aza-CH_3), 24.1 (s, Mes-CH_3), 24.0 (s, Mes-CH_3), 23.6 (s, Aza-CH_3), 23.5 (s, Mes-CH_3), 23.3 (s, Mes-CH_3), 21.4 (s, Mes-CH_3), 21.3 (s, Mes-CH_3), 18.0 (s, Aza-CH_3) ppm.

^{11}B NMR (160.5 MHz, C_6D_6, 298 K): δ = 38.4 (br s, 2 B) ppm.

HRMS (ASAP, $C_{37}H_{52}B_2N_2$): *calcd*: m/z = 546.4311; *found*: m/z = 546.4319.

Elemental analysis: % *calcd*: C 81.33, H 9.59, N 5.13; *found*: C 81.01, H 9.86, N 4.89.

Synthesis of 4,5'-bi-1,2-azaborinine **10**

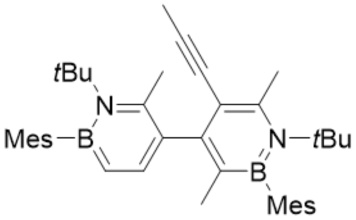

The 1-rhoda-2-mesityl-3-*tert*-butyl-4-methyl-5-(prop-1-ynyl)-3,2-azaborole complex **9**[1] (100 mg, 176 µmol) and 1-*tert*-butyl-2-mesityl-6-methyl-5-(prop-1-ynyl)-1,2-azaborinine (**7**)[1] (41.2 mg, 135 µmol) were dissolved in benzene (8 mL) and the reaction mixture was stirred for 4 d at 84 °C. After removing all volatiles *in vacuo*, the residue was purified by column chromatography on silica gel with a mixture of hexane and diethylether (50:1) as eluent. Evaporation of the solvent from the first fractions yielded a colorless, crystalline solid. The solid was washed with pentane (3 x 0.5 mL) at −30 °C and dried under reduced pressure to yield **10** as a colorless, crystalline solid (30.0 mg, 51.3 µmol, 38%). Crystals of **10** suitable for X-ray diffraction were obtained by evaporation of a saturated hexane solution.

^{1}H NMR (500.1 MHz, C_6D_6, 298 K): δ = 7.40 (d, $^3J_{HH}$ = 10.8 Hz, 1H, Aza-C*H*), 6.92 (s, 1H, Mes-C*H*), 6.89 (s, 1H, Mes-C*H*), 6.88 (s, 1H, Mes-C*H*), 6.87 (s, 1H, Mes-C*H*), 6.67 (d,

$^3J_{HH}$ = 10.8 Hz, 1H, Aza-C*H*), 2.89 (s, 3H, Aza-CH_3), 2.50 (s, 3H, Aza-CH_3), 2.34 (s, 3H, Mes-CH_3), 2.29 (m, 6H, Mes-CH_3), 2.26 (s, 3H, Mes-CH_3), 2.25 (s, 3H, Mes-CH_3), 2.24 (s, 3H, Mes-CH_3), 1.75 (s, 6H, Aza-CH_3 overlapping with CCCH_3), 1.46 (s, 9H, *t*Bu-CH_3), 1.37 (s, 9H, *t*Bu-CH_3) ppm.

^{13}C{^{1}H} NMR (125.8 MHz, C_6D_6, 298 K): δ = 156.2 (s, Aza-C_q), 148.1 (br s, Mes-C_q), 148.0 (s, Aza-C_q), 146.3 (br s, Mes-C_q), 143.9 (s, Aza-*C*H), 141.9 (s, Aza-C_q), 137.2 (s, Mes-C_q), 137.1 (s, Mes-C_q), 136.9 (s, Mes-C_q), 136.8 (s, Mes-C_q), 135.7 (s, Mes-C_q), 135.22 (s, Mes-C_q), 135.16 (br s, Aza-C_q), 131.7 (br s, Aza-*C*H), 128.9 (s, Aza-C_q), 128.0 (s, Mes-*C*H), 127.9 (detected by HSQC, Mes-*C*H), 127.8 (s, Mes-*C*H), 127.7 (s, Mes-*C*H), 112.9 (s, Aza-C_q), 91.0 (s, C*C*CH_3), 80.5 (s, *C*CCH_3), 61.8 (s, *t*Bu-C_q), 60.8 (s, *t*Bu-C_q), 35.0 (s, *t*Bu-*C*H_3), 34.9 (s, *t*Bu-*C*H_3), 25.8 (s, Aza-*C*H_3), 24.1 (s, Mes-*C*H_3), 23.9 (s, Mes-*C*H_3), 23.5 (s, Mes-*C*H_3), 23.32 (s, Mes-*C*H_3), 23.27 (s, Aza-*C*H_3), 21.40 (s, Mes-*C*H_3), 21.38 (s, Mes-*C*H_3), 18.3 (s, Aza-*C*H_3), 4.5 (s, CC*C*H_3) ppm.

^{11}B NMR (160.5 MHz, C_6D_6, 298 K): δ = 38.4 (br s, 2 B) ppm.

HRMS (ASAP, $C_{40}H_{54}B_2N_2$): *calcd*: m/z = 584.4468; *found*: m/z = 584.4482.

Elemental analysis: % *calcd*: C 82.20, H 9.31, N 4.79; *found*: C 82.20, H 9.57, N 4.76.

Synthesis of *p*-phenylene-bridged bis-1,2-azaborinine **12**

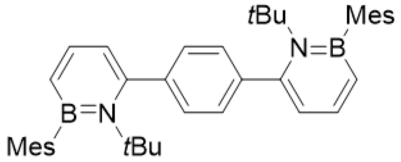

The dirhodium bis(azaborete) complex **11**[1] (120 mg, 107 µmol) was dissolved in benzene (15 mL) and the argon atmosphere was replaced by acetylene. After stirring the reaction mixture for 2 h at 87 °C, the atmosphere was replaced again by acetylene and the reaction mixture was stirred for further 1.5 h at 87 °C. All volatiles were removed *in vacuo* and the residue was purified by column chromatography on silica gel with a mixture of pentane and dichloromethane (10:1) as eluent. Evaporation of the product-containing fractions afforded a colorless solid. The solid was washed with pentane (3 x 1.5 mL) and dried under reduced pressure to yield bis-1,2-azaborinine **12** as a colorless solid (15.0 mg, 25.8 µmol, 24%). Crystals of **12** suitable for X-ray diffraction were obtained by evaporation of a saturated pentane/diethyl ether solution at −30 °C.

^{1}H NMR (500.1 MHz, C_6D_6, 298 K): δ = 7.42-7.38 (m, 2H, Aza-C*H*), 7.31-7.30 (m, 4H, C_6H_4-C*H*), 6.94 (s, 4H, Mes-C*H*), 6.79 (dd, $^{4}J_{HH}$ = 1.8 Hz, $^{3}J_{HH}$ = 10.6 Hz, 2H, Aza-C*H*), 6.18 (dd, $^{4}J_{HH}$ = 1.6 Hz, $^{3}J_{HH}$ = 6.2 Hz, 1H, Aza-C*H*), 6.13 (dd, $^{4}J_{HH}$ = 1.7 Hz, $^{3}J_{HH}$ = 6.2 Hz, 1H, Aza-C*H*), 2.42 (s, 12H, Mes-CH_3), 2.29 (s, 6H, Mes-CH_3), 1.35 (s, 9H, *t*Bu-CH_3), 1.33 (s, 9H, *t*Bu-CH_3) ppm.

^{13}C{^{1}H} NMR (125.8 MHz, C_6D_6, 298 K): δ = 151.01 (s, Aza-C_q), 150.98 (s, Aza-C_q), 146.0 (br s, Mes-C_q), 144.1 (s, C_6H_4-C_q), 144.0 (s, C_6H_4-C_q), 141.2 (s, Aza-*C*H), 138.1 (s, Mes-C_q), 137.9 (s, Mes-C_q), 136.30 (s, Mes-C_q), 136.23 (s, Mes-C_q), 133.7 (br s, Aza-*C*H), 129.0 (s, C_6H_4-*C*H), 128.7 (s, C_6H_4-*C*H), 128.1 (detected by HSQC, Mes-*C*H), 119.4 (s, Aza-*C*H), 119.1 (s, Aza-*C*H), 62.70 (s, *t*Bu-C_q), 62.63 (s, *t*Bu-C_q), 34.7 (s, *t*Bu-CH_3), 34.5 (s, *t*Bu-CH_3), 24.32 (s, Mes-CH_3), 24.29 (s, Mes-CH_3), 21.3 (s, Mes-CH_3) ppm.

^{11}B NMR (160.5 MHz, C_6D_6, 298 K): δ = 40.8 (br s) ppm.

HRMS (ASAP, $C_{40}H_{50}B_2N_2$ + H): *calcd*: m/z = 581.4233; *found*: m/z = 581.4212.

Elemental analysis: % *calcd*: C 82.77, H 8.68, N 4.83; *found*: C 83.13, H 8.82, N 4.89.

NMR Spectra

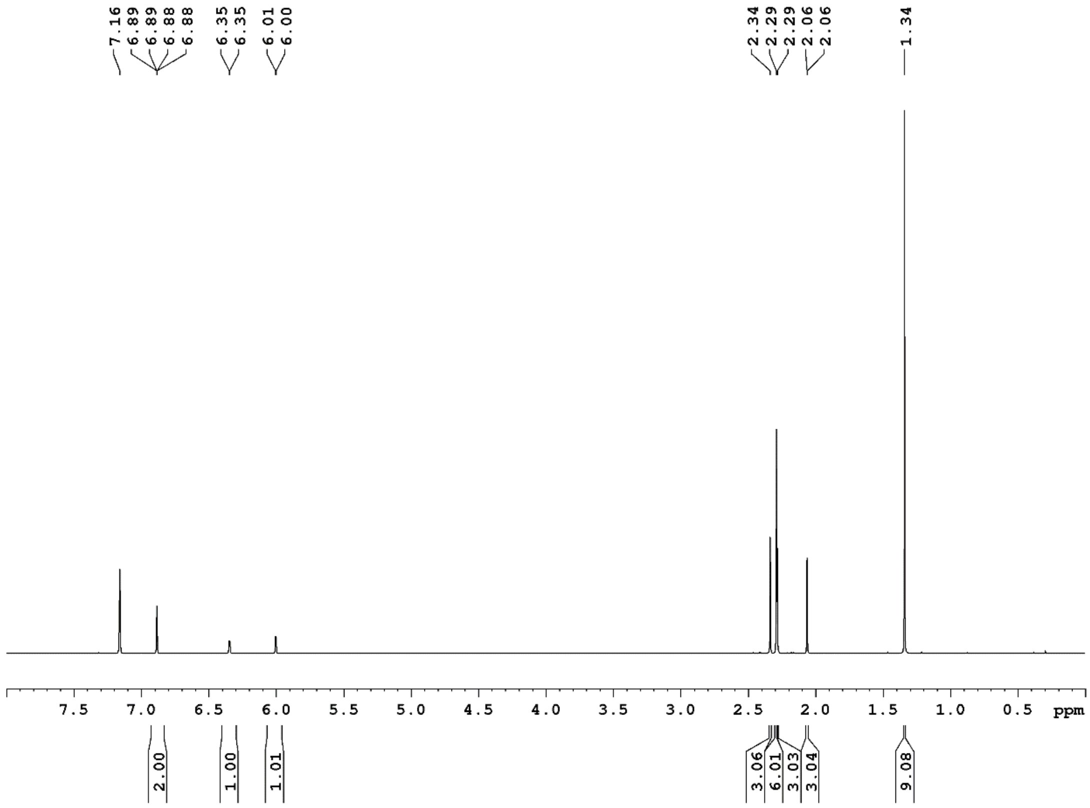

Figure S1. ^{1}H NMR (500.1 MHz, C_6D_6, 298 K) spectrum of 1-*tert*-butyl-2-mesityl-4,6-dimethyl-1,2-azaborinine (**2**).

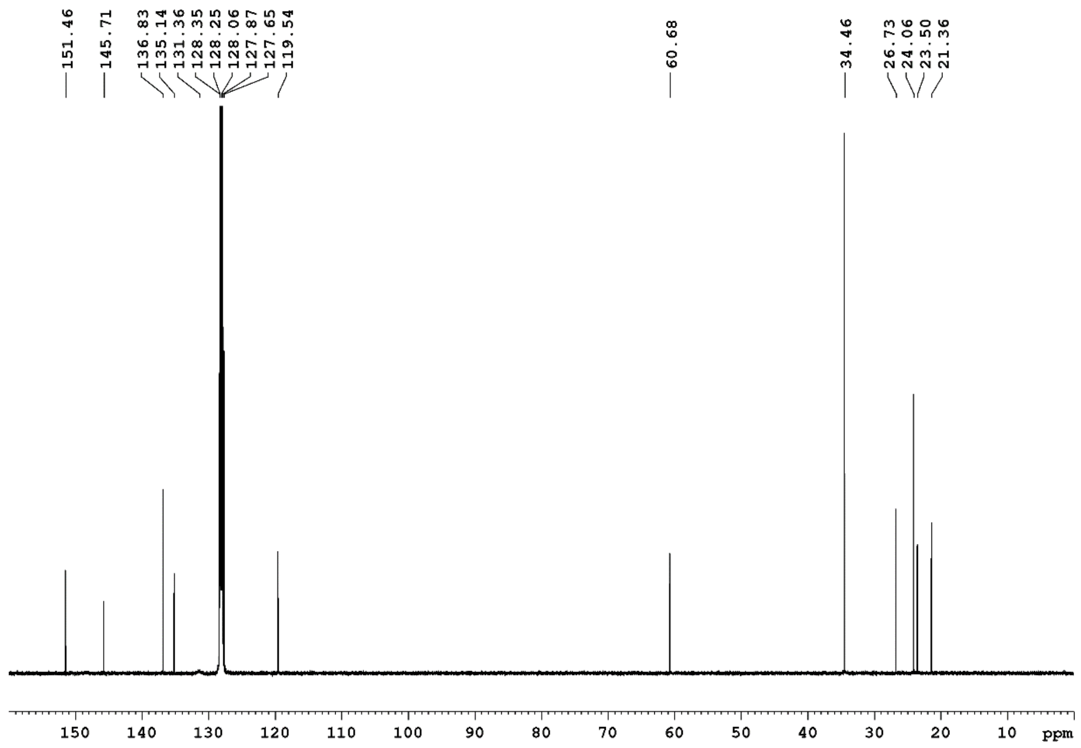

Figure S2. $^{13}C\{^{1}H\}$ NMR (125.8 MHz, C_6D_6, 298 K) spectrum of 1-*tert*-butyl-2-mesityl-4,6-dimethyl-1,2-azaborinine (**2**).

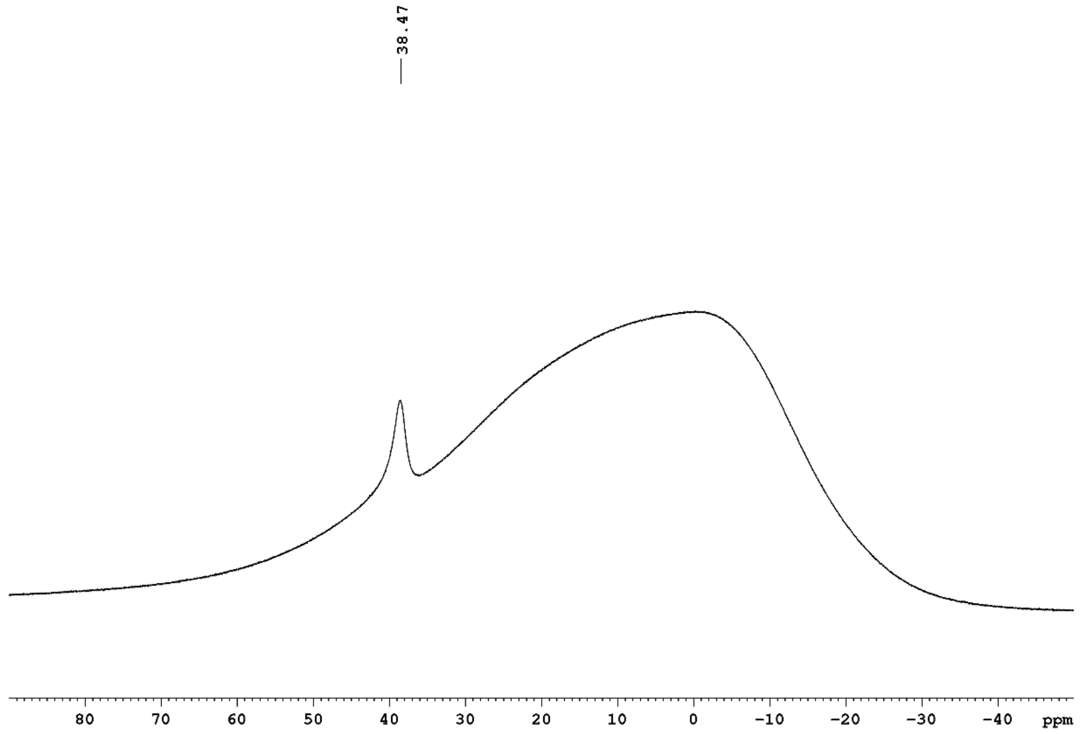

Figure S3. ^{11}B NMR (160.5 MHz, C_6D_6, 298 K) spectrum of 1-*tert*-butyl-2-mesityl-4,6-dimethyl-1,2-azaborinine (**2**).

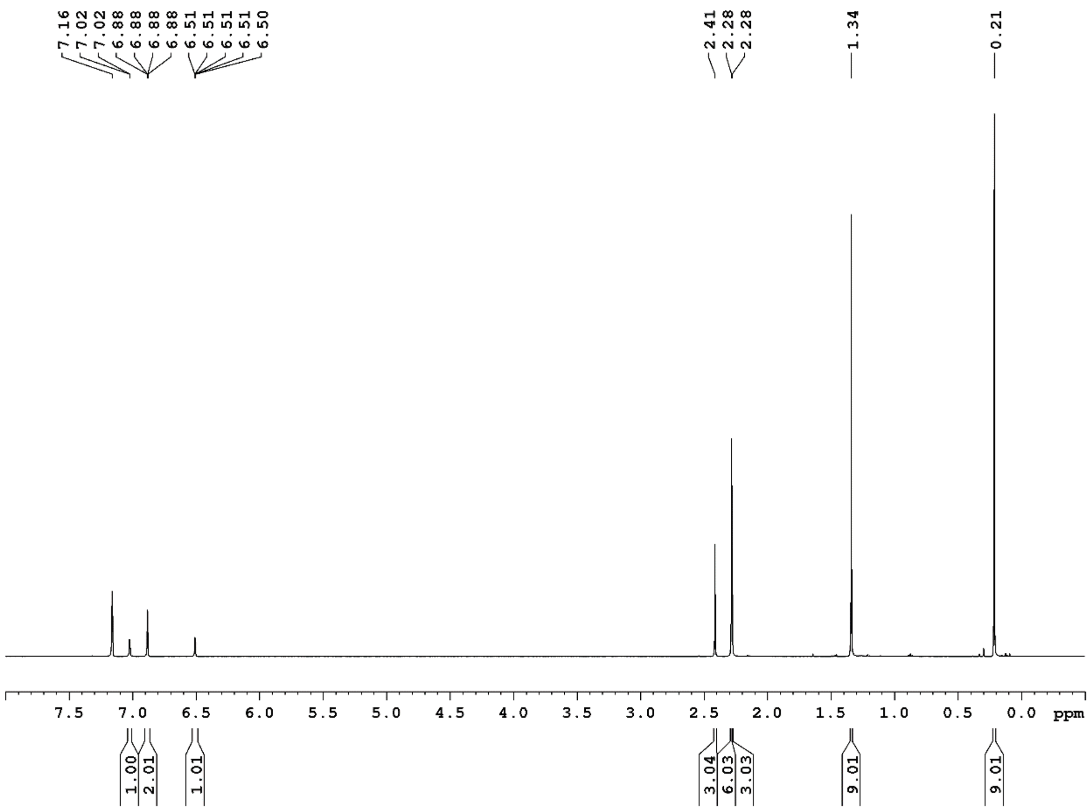

Figure S4. ^{1}H NMR (500.1 MHz, C_6D_6, 298 K) spectrum of 1-*tert*-butyl-2-mesityl-6-methyl-4-trimethylsilyl-1,2-azaborinine (**3**).

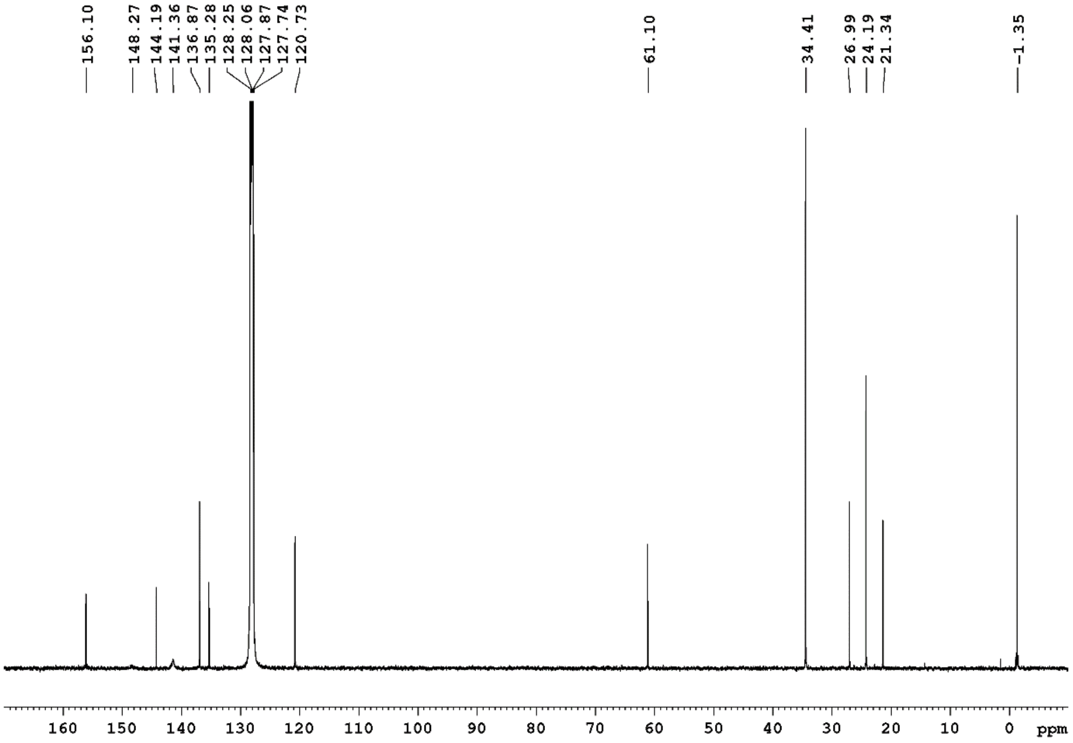

Figure S5. $^{13}C\{^{1}H\}$ NMR (125.8 MHz, C_6D_6, 298 K) spectrum of 1-*tert*-butyl-2-mesityl-6-methyl-4-trimethylsilyl-1,2-azaborinine (**3**).

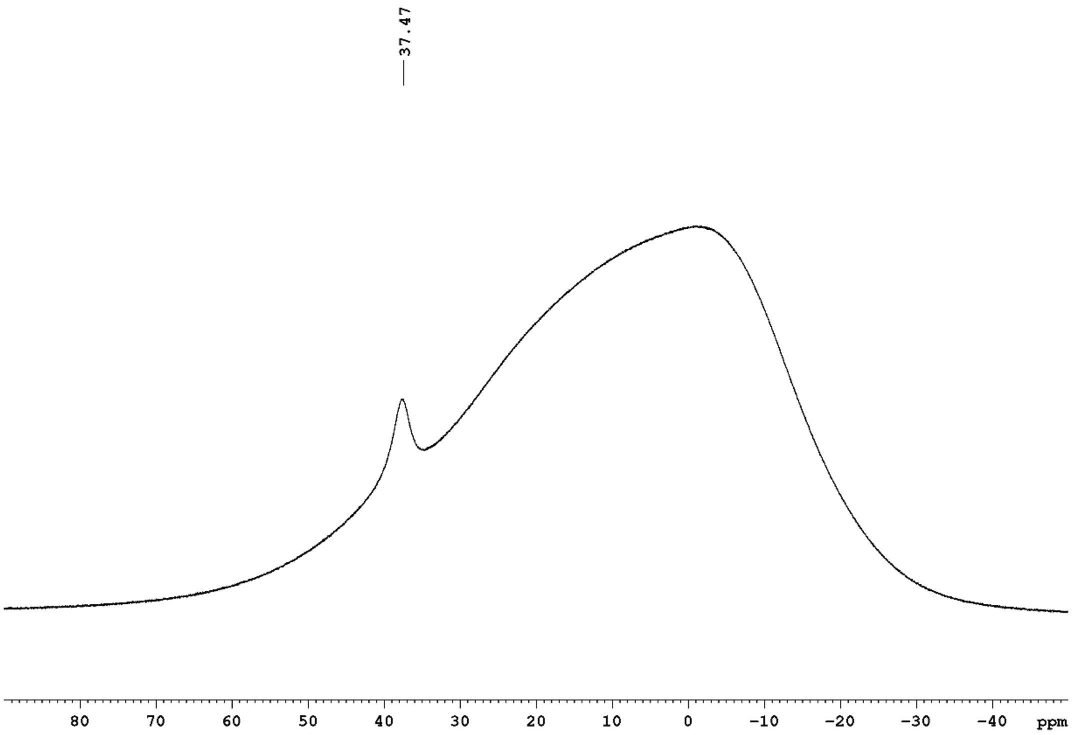

Figure S6. ^{11}B NMR (160.5 MHz, C_6D_6, 298 K) spectrum of 1-*tert*-butyl-2-mesityl-6-methyl-4-trimethylsilyl-1,2-azaborinine (**3**).

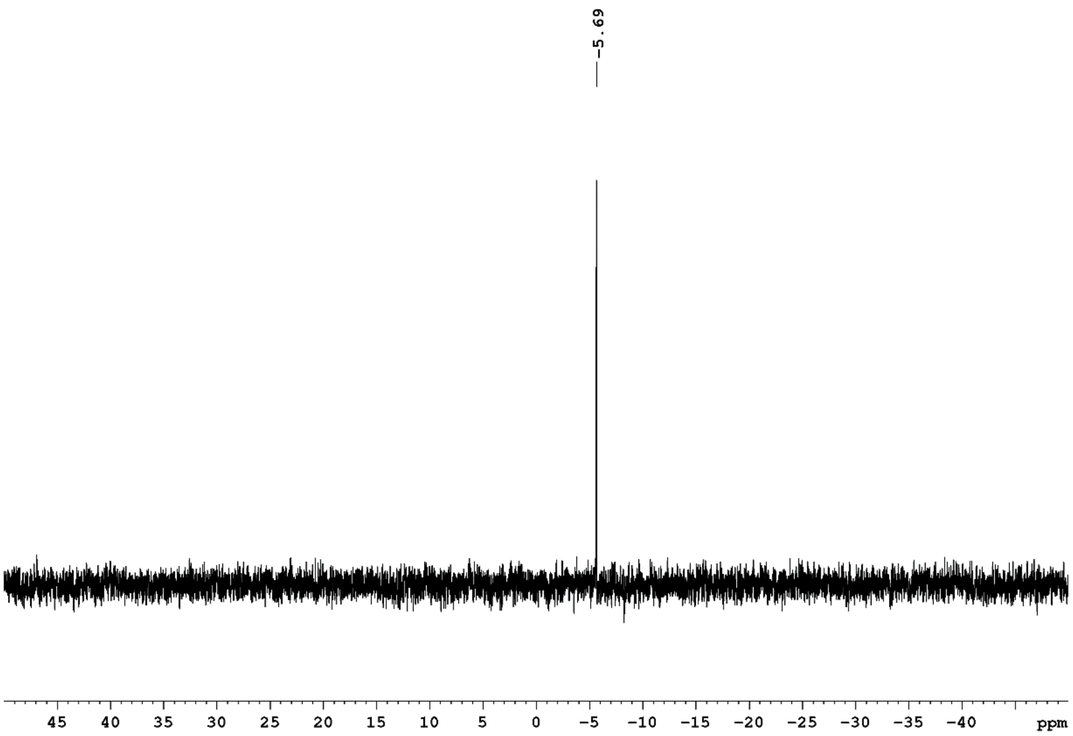

Figure S7. ^{29}Si NMR (99.4 MHz, C_6D_6, 298 K) spectrum of 1-*tert*-butyl-2-mesityl-6-methyl-4-trimethylsilyl-1,2-azaborinine (**3**).

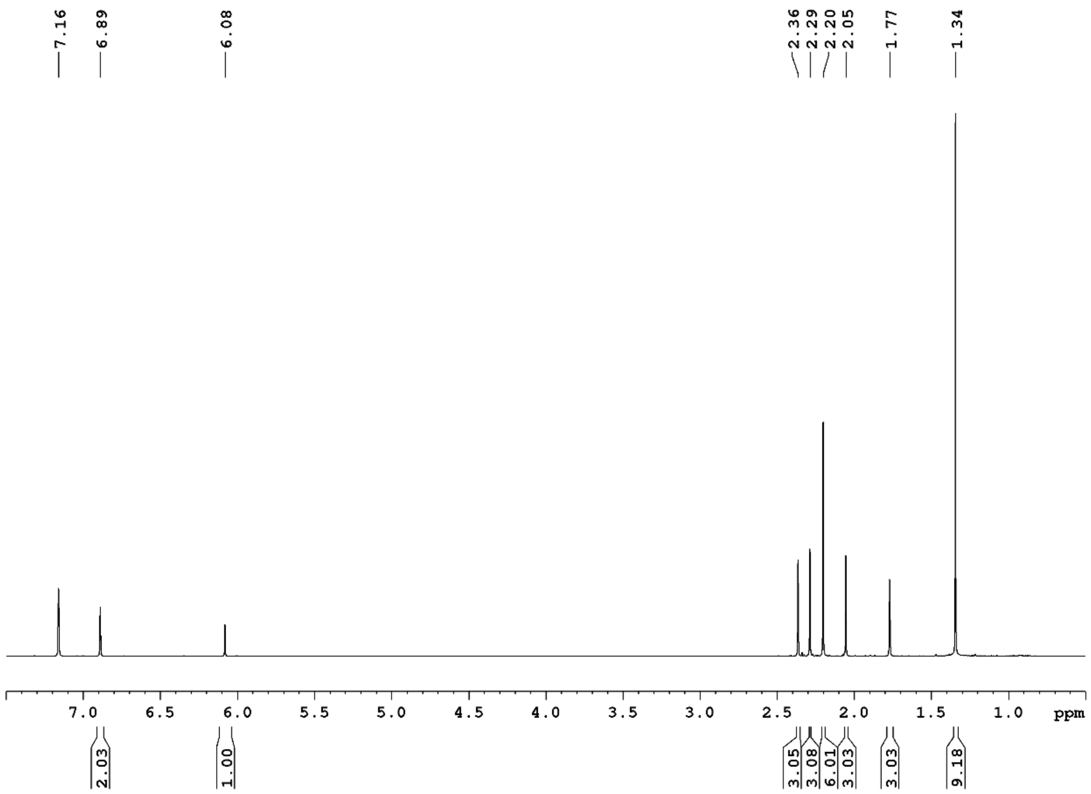

Figure S8. ^{1}H NMR (500.1 MHz, C_6D_6, 298 K) spectrum of 1-*tert*-butyl-2-mesityl-3,4,6-trimethyl-1,2-azaborinine (**4**).

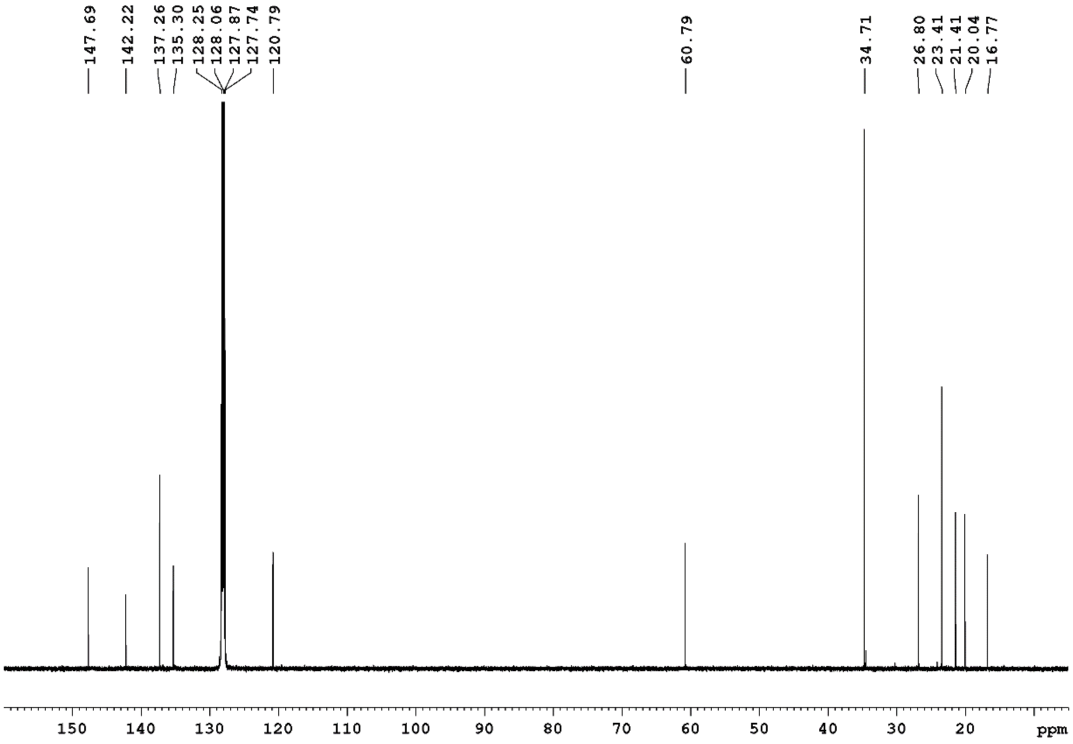

Figure S9. $^{13}C\{^{1}H\}$ NMR (125.8 MHz, C_6D_6, 298 K) spectrum of 1-*tert*-butyl-2-mesityl-3,4,6-trimethyl-1,2-azaborinine (**4**).

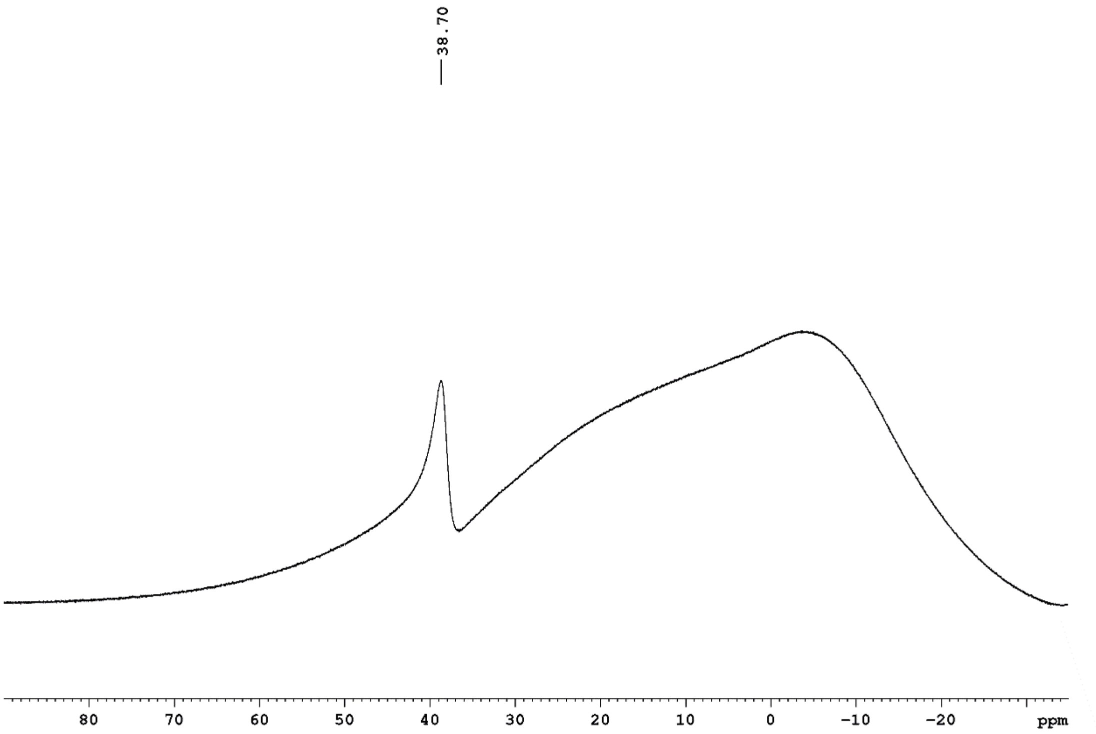

Figure S10. ^{11}B NMR (160.5 MHz, C_6D_6, 298 K) spectrum of 1-*tert*-butyl-2-mesityl-3,4,6-trimethyl-1,2-azaborinine (**4**).

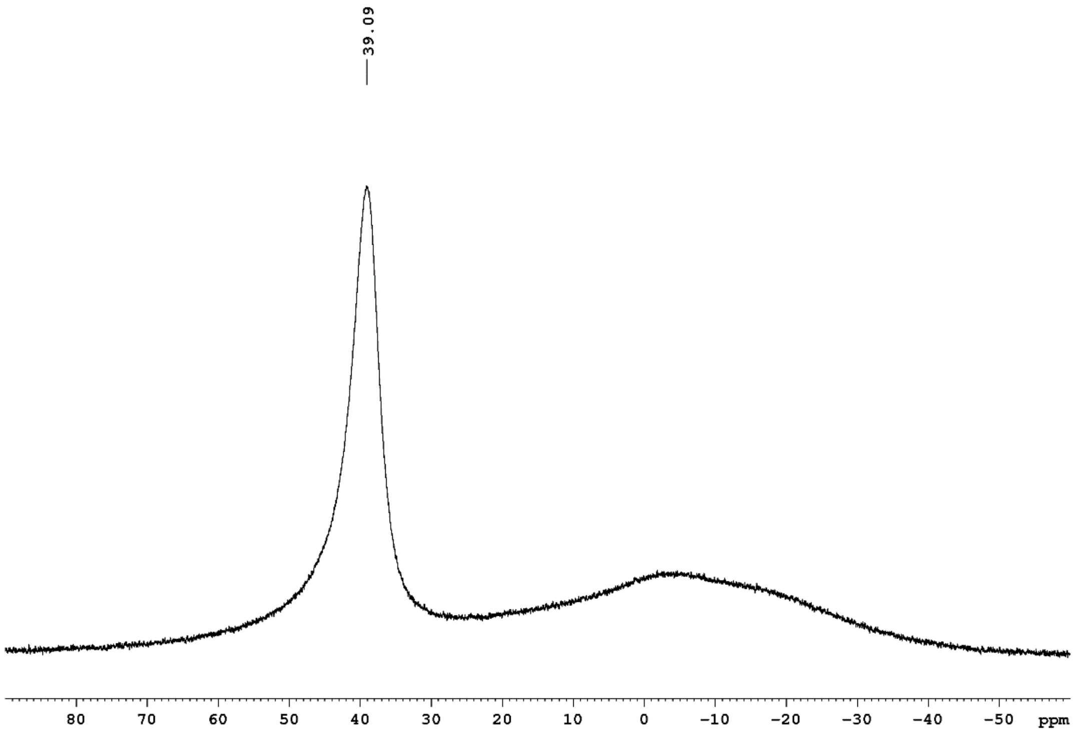

Figure S11. ^{11}B NMR (128.4 MHz, C_6D_6, 298 K) spectrum of 1-*tert*-butyl-3-ethyl-2-mesityl-6-methyl-4-phenyl-1,2-azaborinine (**5**).

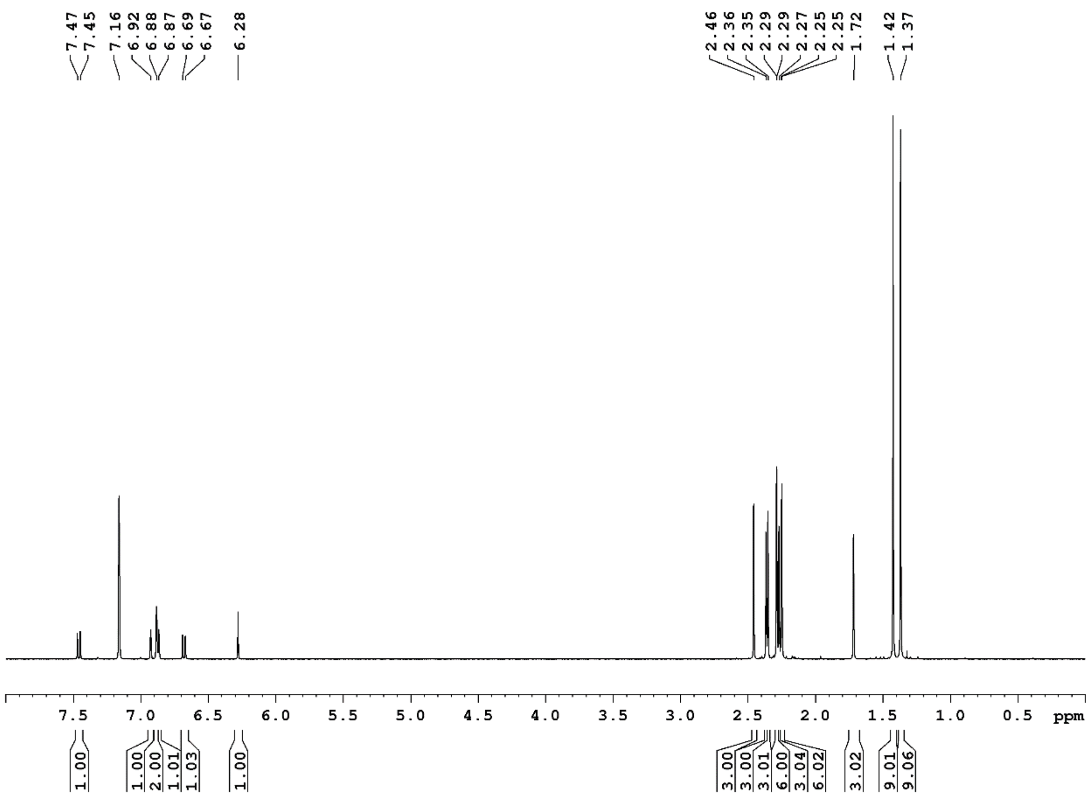

Figure S12. ^{1}H NMR (500.1 MHz, C_6D_6, 298 K) spectrum of 4,5'-bi(azaborinine) **8**.

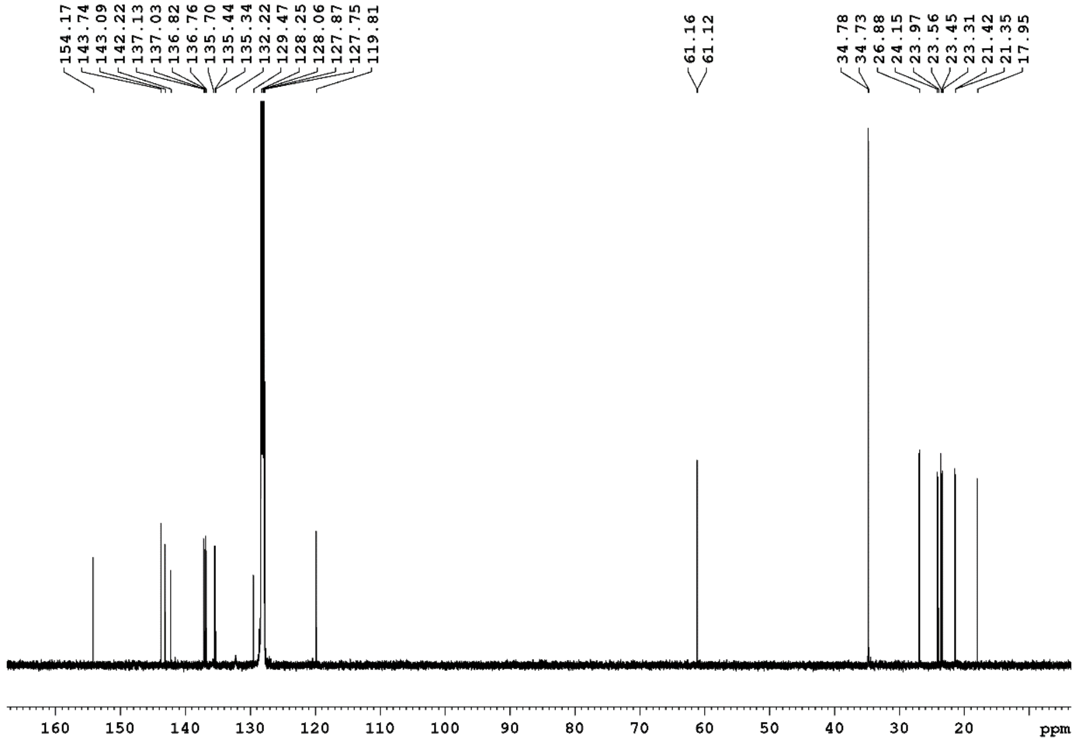

Figure S13. $^{13}C\{^{1}H\}$ NMR (125.8 MHz, C_6D_6, 298 K) spectrum of 4,5'-bi(azaborinine) **8**.

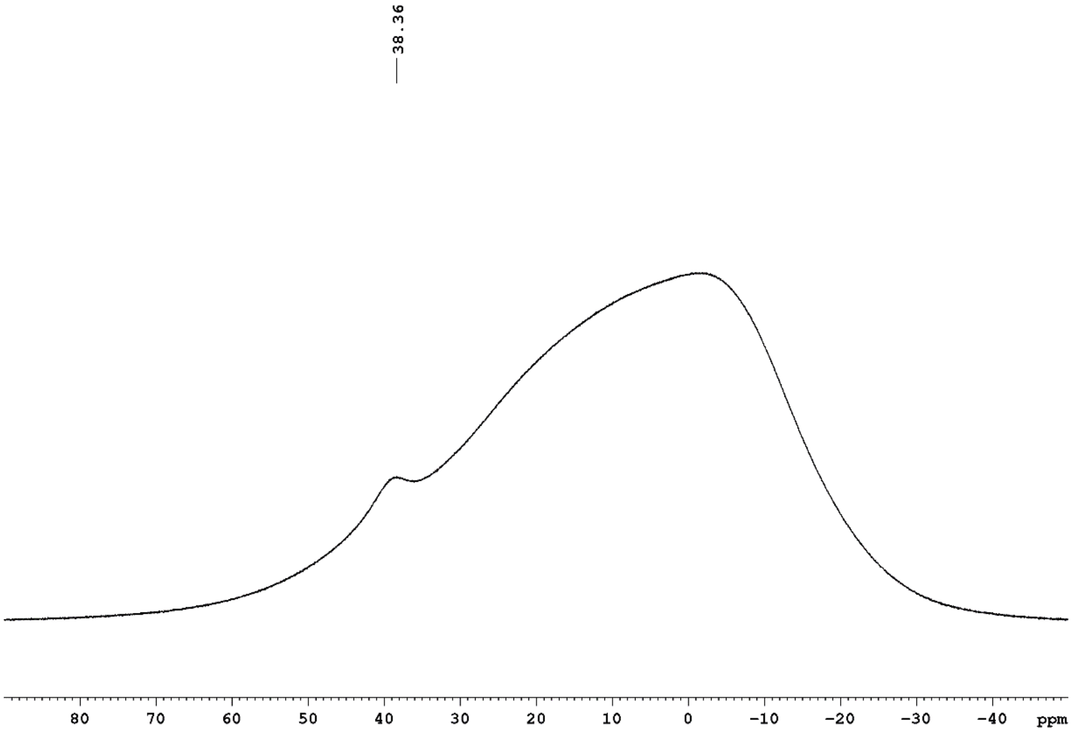

Figure S14. ^{11}B NMR (160.5 MHz, C_6D_6, 298 K) spectrum of 4,5'-bi(azaborinine) **8**.

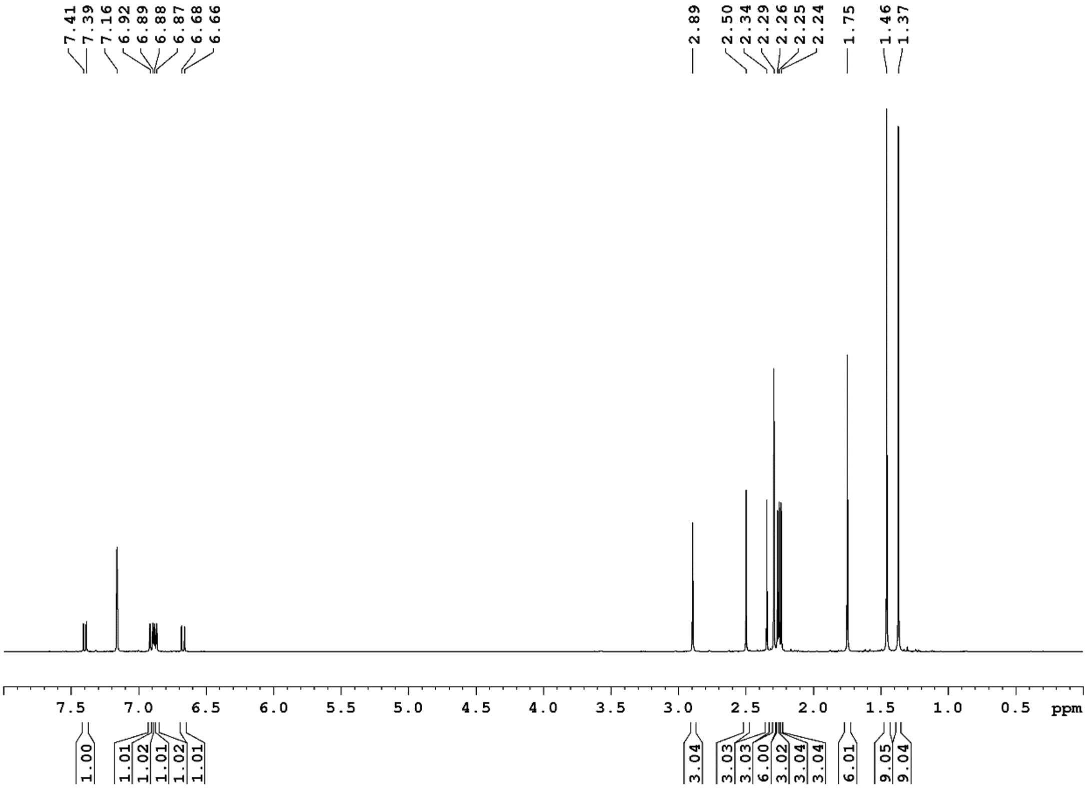

Figure S15. ^{1}H NMR (500.1 MHz, C_6D_6, 298 K) spectrum of 4,5'-bi(azaborinine) **10**.

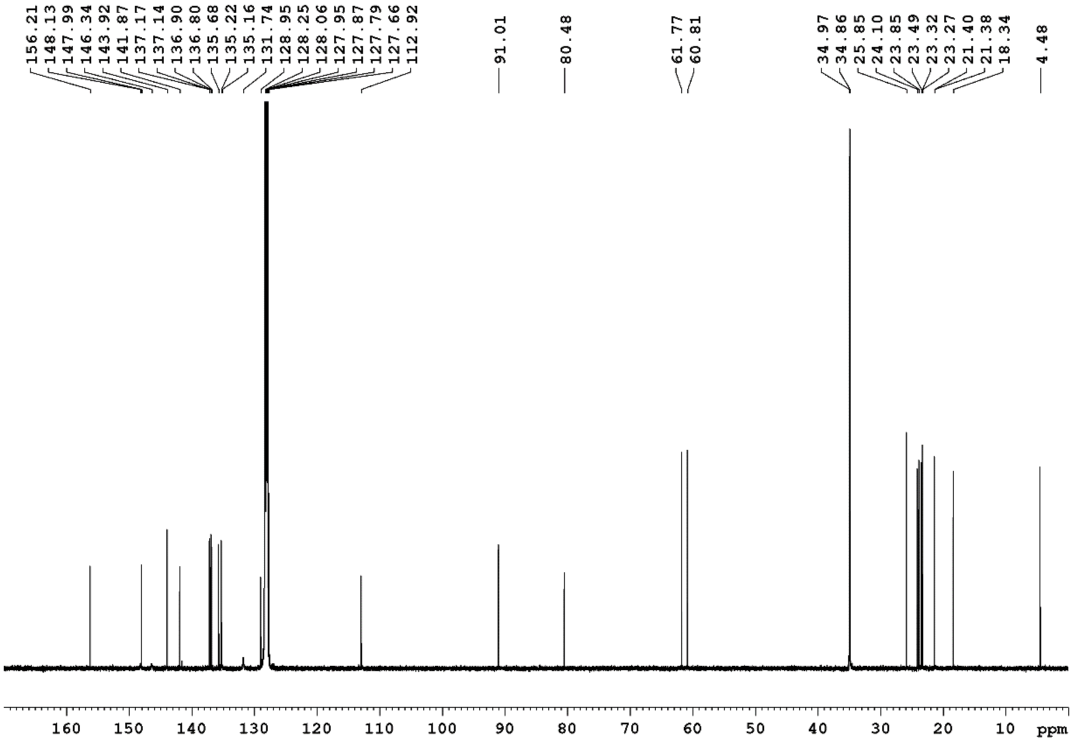

Figure S16. $^{13}C\{^{1}H\}$ NMR (125.8 MHz, C_6D_6, 298 K) spectrum of 4,5’-bi(azaborinine) **10**.

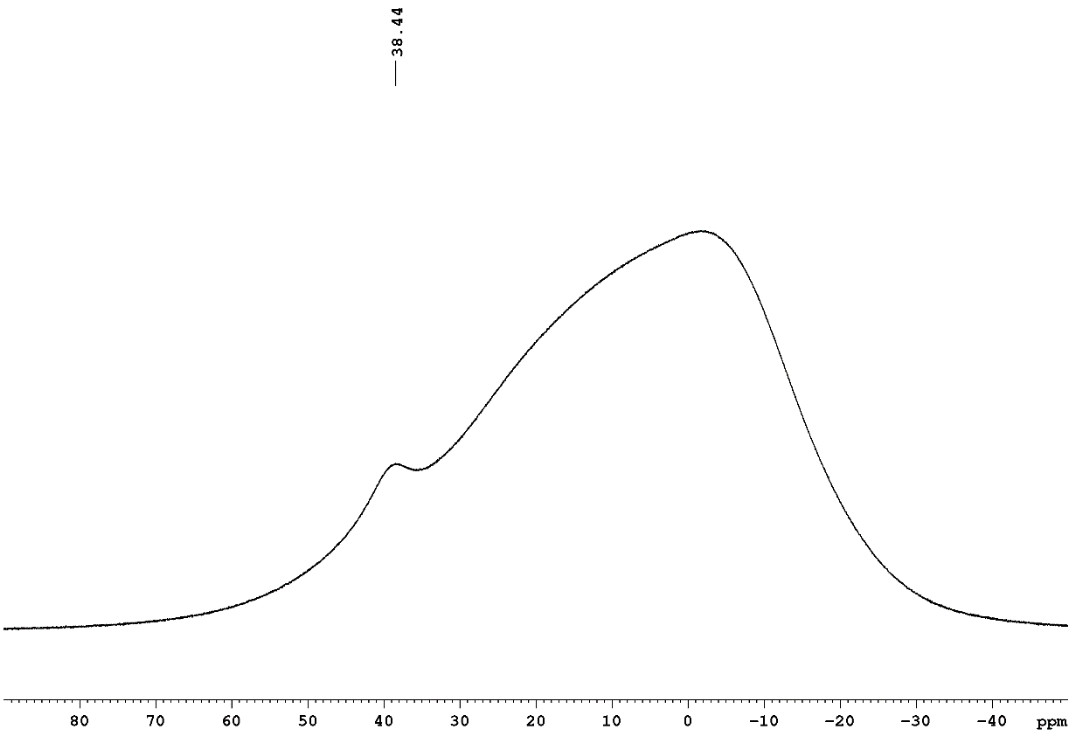

Figure S17. ^{11}B NMR (160.5 MHz, C_6D_6, 298 K) spectrum of 4,5’-bi(azaborinine) **10**.

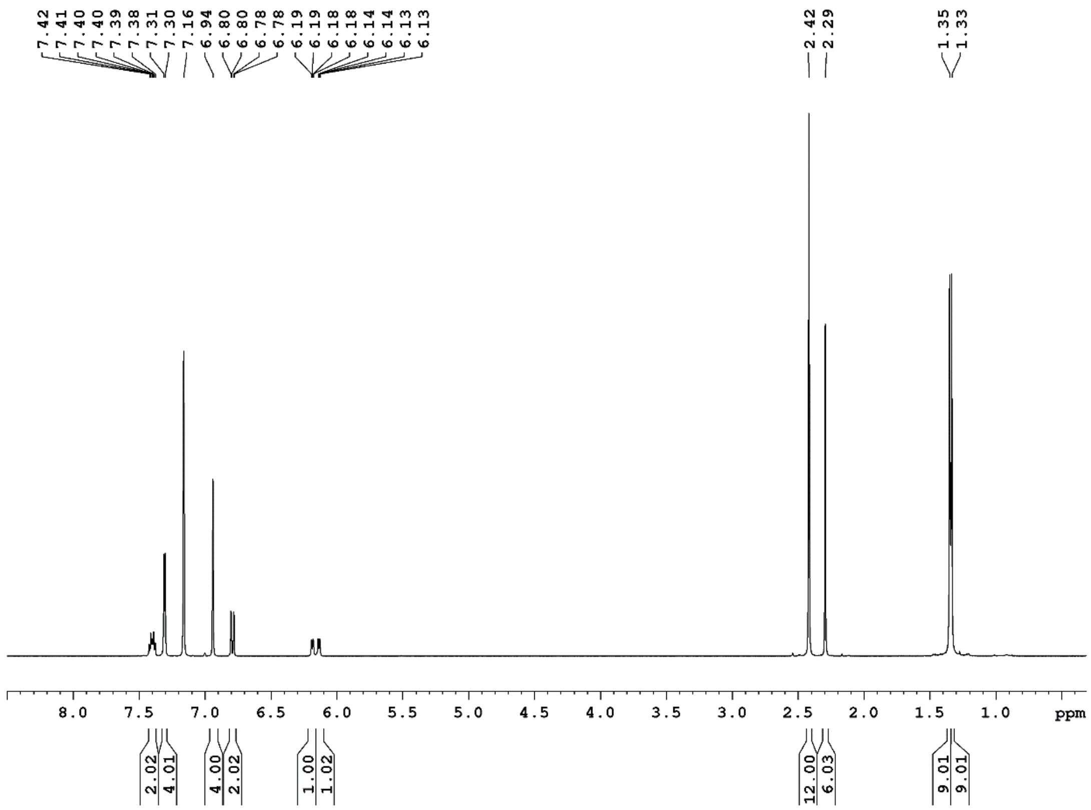

Figure S18. ^{1}H NMR (500.1 MHz, C_6D_6, 298 K) spectrum of 1,4-bis(azaborinine)benzene **12**.

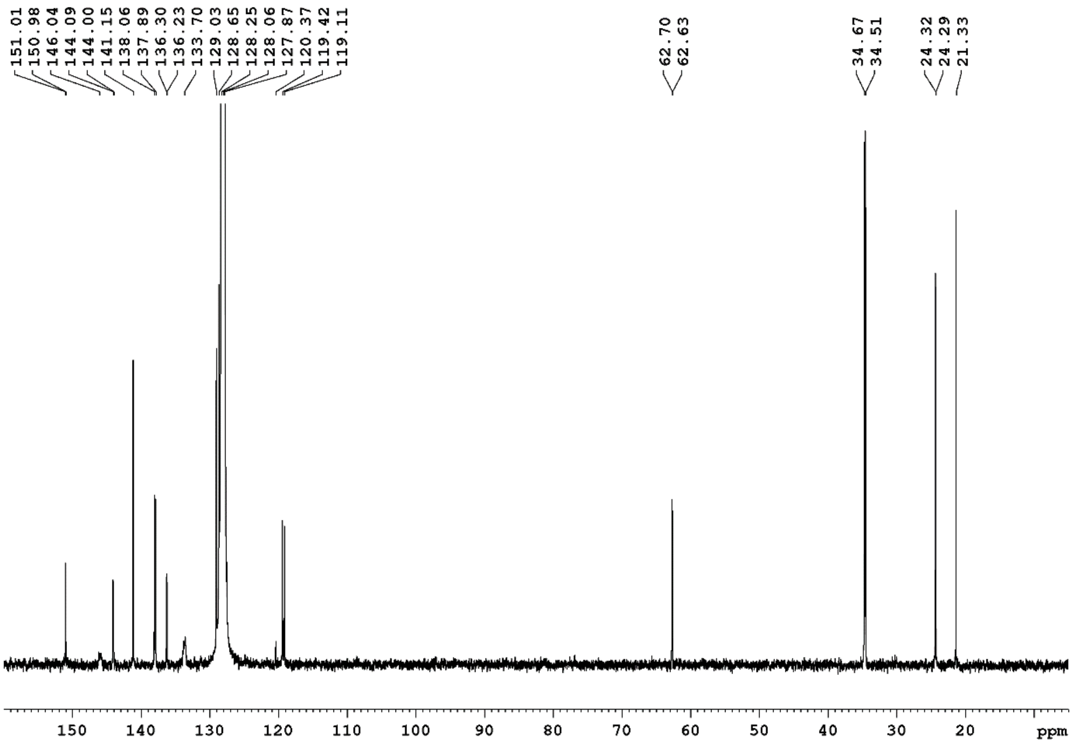

Figure S19. $^{13}C\{^{1}H\}$ NMR (125.8 MHz, C_6D_6, 298 K) spectrum of 1,4-bis(azaborinine)benzene **12**.

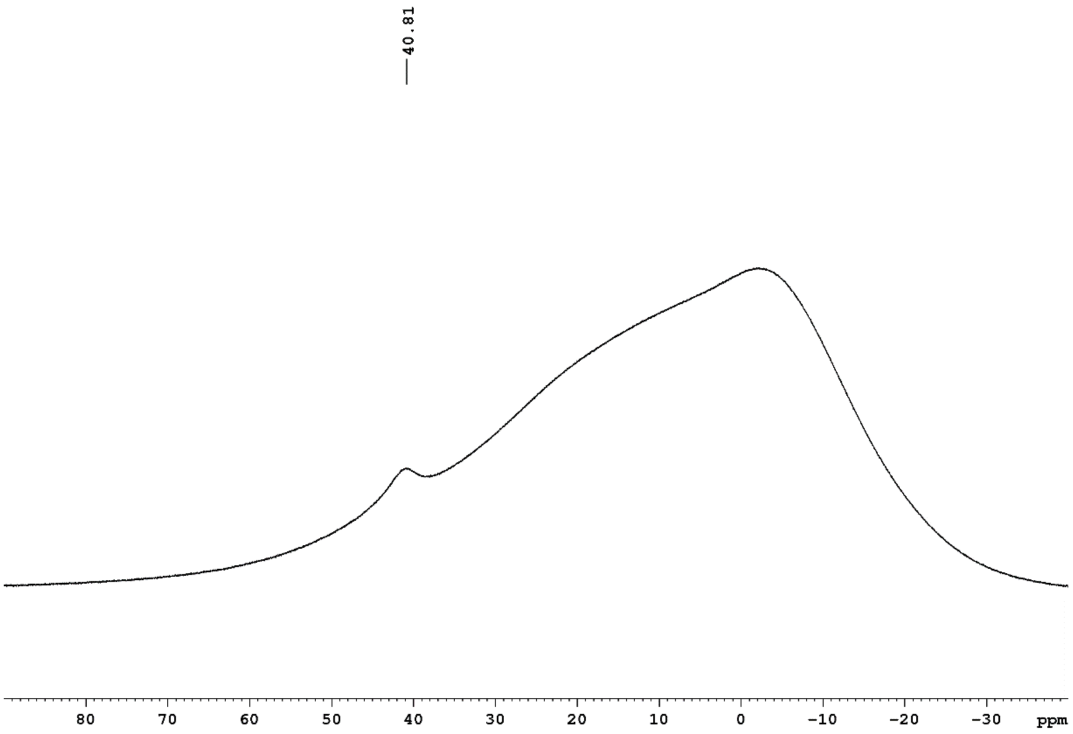

Figure S20. ^{11}B NMR (160.5 MHz, C_6D_6, 298 K) spectrum of 1,4-bis(azaborinine)benzene **12**.

Crystal Structure Determination

The crystal data of 1-*tert*-butyl-2-mesityl-6-methyl-4-trimethylsilyl-1,2-azaborinine (**3**) and the 4,5'-bi-1,2-azaborinines **8** and **10** were collected on a BRUKER D8 QUEST diffractometer with a CMOS area detector and multi-layer mirror monochromated $Mo_{K\alpha}$ radiation. The crystal data of 1-*tert*-butyl-2-mesityl-4,6-dimethyl-1,2-azaborinine (**2**), 1-*tert*-butyl-2-mesityl-3,4,6-trimethyl-1,2-azaborinine (**4**), 1-*tert*-butyl-3-ethyl-2-mesityl-6-methyl-4-phenyl-1,2-azaborinine (**5**), and 1,4-bis(azaborinine)benzene **12** were collected on a BRUKER X8-APEX II diffractometer with a CCD area detector and multi-layer mirror monochromated $Mo_{K\alpha}$ radiation. The structures were solved using the intrinsic phasing method,[4] refined with the SHELXL program[5] and expanded using Fourier techniques. All non-hydrogen atoms were refined anisotropically. Hydrogen atoms were included in structure factor calculations. All hydrogen atoms were assigned to idealized geometric positions.

Crystallographic data have been deposited with the Cambridge Crystallographic Data Center as supplementary publication nos. CCDC 2067502 (**2**), 2067503 (**5**), 2067504 (**12**), 2067505 (**10**), 2067506 (**3**), 2067507 (**8**), and 2067508 (**4**). These data can be obtained free of charge from The Cambridge Crystallographic Data Centre *via* www.ccdc.cam.ac.uk/data_request/cif

Refinement details for **2:** The crystal was a pseudo-merohedral twin with domains rotated by 179.6° around the real axis [-0.174 1.000 -0.035]. The BASF parameter was refined to 31.2%.

Refinement details for **5:** The BUMP instruction was used to avoid short intramolecular H-H contacts.

Refinement details for **8:** The most disagreeable reflection was omitted.

Refinement details for **10:** The BUMP instruction was used to avoid short intramolecular H-H contacts.

Crystal data for **2**: $C_{19}H_{28}BN$, M_r = 281.23, colorless block, 0.318×0.216×0.125 mm^3, triclinic space group $P\bar{1}$, a = 8.1330(3) Å, b = 8.7821(4) Å, c = 12.5256(5) Å, α = 84.912(2)°, β = 75.1850(10)°, γ = 79.871(2)°, V = 850.52(6) Å^3, Z = 2, ρ_{calcd} = 1.098 g·cm^{-3},

$\mu = 0.062$ mm^{-1}, $F(000) = 308$, $T = 100(2)$ K, $R_1 = 0.0719$, $wR^2 = 0.1301$, 3348 independent reflections [2θ≤52.042°] and 210 parameters.

Crystal data for **3**: $C_{21}H_{34}BNSi$, $M_r = 339.39$, colorless block, 0.267×0.191×0.19 mm^3, triclinic space group $P\bar{1}$, $a = 10.598(3)$ Å, $b = 10.675(2)$ Å, $c = 11.998(4)$ Å, $\alpha = 91.088(13)°$, $\beta = 115.78(2)°$, $\gamma = 117.514(12)°$, $V = 1042.1(5)$ Å^3, $Z = 2$, $\rho_{calcd} = 1.082$ g·cm^{-3}, $\mu = 0.115$ mm^{-1}, $F(000) = 372$, $T = 100(2)$ K, $R_1 = 0.0465$, $wR^2 = 0.0885$, 4107 independent reflections [2θ≤52.114°] and 227 parameters.

Crystal data for **4**: $C_{20}H_{30}BN$, $M_r = 295.26$, colorless block, 0.174×0.162×0.077 mm^3, orthorhombic space group $P2_12_12_1$, $a = 9.341(6)$ Å, $b = 9.520(6)$ Å, $c = 19.865(12)$ Å, $V = 1766.4(18)$ Å^3, $Z = 4$, $\rho_{calcd} = 1.110$ g·cm^{-3}, $\mu = 0.062$ mm^{-1}, $F(000) = 648$, $T = 296(2)$ K, $R_1 = 0.0582$, $wR^2 = 0.1121$, 3497 independent reflections [2θ≤52.744°] and 208 parameters.

Crystal data for **5**: $C_{26}H_{34}BN$, $M_r = 371.35$, colorless block, 0.311×0.099×0.051 mm^3, monoclinic space group $C2/c$, $a = 23.351(18)$ Å, $b = 14.265(13)$ Å, $c = 15.830(13)$ Å, $\beta = 121.46(3)°$, $V = 4498(6)$ Å^3, $Z = 8$, $\rho_{calcd} = 1.097$ g·cm^{-3}, $\mu = 0.062$ mm^{-1}, $F(000) = 1616$, $T = 103(2)$ K, $R_1 = 0.1377$, $wR^2 = 0.1484$, 4399 independent reflections [2θ≤52.042°] and 261 parameters.

Crystal data for **8**: $C_{37}H_{52}B_2N_2$, $M_r = 546.42$, colorless block, 0.267×0.165×0.108 mm^3, monoclinic space group $P2_1/c$, $a = 18.112(5)$ Å, $b = 14.3222(19)$ Å, $c = 13.0892(18)$ Å, $\beta = 99.508(10)°$, $V = 3348.7(11)$ Å^3, $Z = 4$, $\rho_{calcd} = 1.084$ g·cm^{-3}, $\mu = 0.061$ mm^{-1}, $F(000) = 1192$, $T = 100(2)$ K, $R_1 = 0.0554$, $wR^2 = 0.1257$, 6845 independent reflections [2θ≤52.744°] and 385 parameters.

Crystal data for **10**: $C_{40}H_{54}B_2N_2$, $M_r = 584.47$, colorless block, 0.297×0.294×0.151 mm^3, triclinic space group $P\bar{1}$, $a = 7.9083(18)$ Å, $b = 14.762(3)$ Å, $c = 15.828(5)$ Å, $\alpha = 100.907(7)°$, $\beta = 94.289(11)°$, $\gamma = 96.152(9)°$, $V = 1795.4(7)$ Å^3, $Z = 2$,

ρ_{calcd} = 1.081 g·cm^{-3}, μ = 0.061 mm^{-1}, F(000) = 636, T = 100(2) K, R_1 = 0.0556, wR^2 = 0.1259, 7326 independent reflections [2θ≤52.742°] and 413 parameters.

Crystal data for **12**: $C_{40}H_{50}B_2N_2$, M_r = 580.44, colorless plate, 0.406×0.305×0.094 mm^3, triclinic space group $P\bar{1}$, a = 6.811(2) Å, b = 8.306(3) Å, c = 16.501(7) Å, α = 96.823(13)°, β = 92.959(9)°, γ = 112.696(6)°, V = 850.2(5) Å^3, Z = 1, ρ_{calcd} = 1.134 g·cm^{-3}, μ = 0.064 mm^{-1}, F(000) = 314, T = 100(2) K, R_1 = 0.0531, wR^2 = 0.1166, 3490 independent reflections [2θ≤52.74°] and 205 parameters.

References

[1] M. Heß, T. E. Stennett, F. Fantuzzi, R. Bertermann, M. Schock, M. Schäfer, T. Thiess, H. Braunschweig, *Chem. Sci.* **2020**, *11*, 9134-9140.

[2] W. Wolfsberger, H. Schmidbaur, *Syn. React. Inorg. Metal-Org. Chem.* **1974**, *4*, 149-156.

[3] M. Schäfer, J. Schäfer, R. D. Dewhurst, W. C. Ewing, M. Krahfuß, M. W. Kuntze-Fechner, M. Wehner, C. Lambert, H. Braunschweig, *Chem. Eur. J.* **2016**, *22*, 8603-8609.

[4] G. Sheldrick, *Acta Cryst.* **2015**, *A71*, 3-8.

[5] G. Sheldrick, *Acta Cryst.* **2008**, *A64*, 112-122.
